# Amorphous–Crystalline Solid Transformation-Induced Self-Actuation of Bending-to-Straightening Behavior via Helical Deformation

**DOI:** 10.1021/acs.cgd.5c01103

**Published:** 2025-11-05

**Authors:** Xintong Meng, Yifan Huang, Qun Song, Ruhuai Mei, Lin Tian, Corinna Willenberg, Fen Li, Cynthia Volkert, Philipp Vana, Qiyun Tang, Ping Shao, Xun Wang, Kai Zhang

**Affiliations:** † Sustainable Materials and Chemistry, Department of Wood Technology and Wood-based Composites, 9375 University of Göttingen, Büsgenweg 4, Göttingen 37077, Germany; ‡ Key Laboratory of Quantum Materials and Devices of Ministry of Education, School of Physics, 12579 Southeast University, Nanjing 211189, China; § Institute of Materials Physics, University of Göttingen, Friedrich-Hund-Platz. 1, Göttingen 37077, Germany; ∥ Institute of Inorganic Chemistry, University of Göttingen, Tammannstr. 4, Göttingen 37077, Germany; ⊥ Institute of Physical Chemistry, University of Göttingen, Tammannstr. 6, Göttingen 37077, Germany; # Department of Food Science and Technology, Zhejiang University of Technology, Hangzhou 310014, China; ¶ Faculty of Chemistry, 12442 Tsinghua University, Beijing 100084, China; ∇ Wöhler Research Institute of Sustainable Chemistry (WISCh), University of Göttingen, Tammanstr. 2, Göttingen 37077, Germany

## Abstract

Bending-to-straightening behavior is vital for both natural processes and advanced materials design. Nonclassical crystallization pathways, particularly amorphous–crystalline transformations, offer opportunities to achieve such dynamic actuation. This study reveals that sugar azides are capable of undergoing spontaneous bending-to-straightening behavior, accompanied by helical deformation, driven by an amorphous–crystalline transformation during anisotropic self-assembly. The process is initiated with bending amorphous nanowires, which evolve into locally crystallized twisted nanoribbons and ultimately straighten into crystalline rectangular hollow tubes through screw dislocation. This transformation is governed by the interplay between the stereostructure of the sugar backbone and the collinear dipole arrangement of the azide group, which together regulates initial helical aggregation and subsequent directional growth. These findings not only clarify the molecular origins of bending-to-straightening crystallization but also provide a strategy for designing responsive materials capable of adapting to unstructured environments.

## Graphical abstract

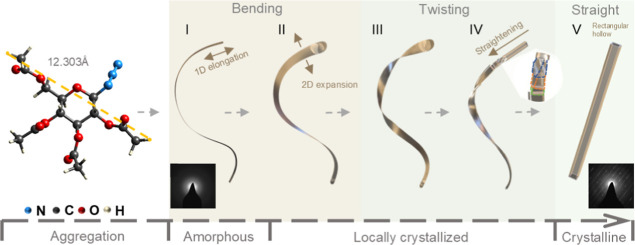

## Introduction

Transforming energy into motion via photo, thermal, humidity, or chemical activation is central to both natural processes and advanced materials design.^1–4^ Bending and twisting are recognized as the two primary actuation forms among the various actuation behaviors in natural examples, such as hypocotyl straightening and twisting of seedpods.^5,6^ Some are closely linked to the chirality of their building blocks, as chirality is a fundamental feature of living matter and natural systems.^7^ Beyond molecular chirality that gives rise to twisting, bending and twisting can also arise from twinning, surface stress, asymmetric crystal growth, or dislocations in crystallography, revealing a structural basis for complex motion at larger scales.^8–11^

Bending-to-straightening behaviors have been increasingly studied in recent years, particularly those triggered by external stimuli, which typically involve reversible processes.^12^ Such externally driven motions are commonly observed in systems such as hydrogels, liquid crystal elastomers, and crystals undergoing solid–solid transitions between polymorphs.^13–15^ These transitions can be initiated by stimuli including hydration, light, heat, electric fields, or mechanical force.^16–18^ Moreover, molecules that tend to form slip planes or layered packing are often favored in such crystals that exhibit structural distortion.^19,20^ However, growth-induced bending-to-straightening behavior remains less studied in materials science and is distinct from externally driven bending-to-straightening behavior. As an irreversible process, growth-induced transitions from bent to straight configurations are fundamental to various biological functions and have inspired research across fields ranging from molecular machines to self-growing robots for navigating, exploring, and colonizing unstructured environments.^21–23^

Amorphous–crystalline transformation, as a representative nonclassical crystallization pathway, involves the formation of an amorphous intermediate that serves as a precursor to crystal growth and has been experimentally confirmed in a wide range of systems, particularly in two-step nucleation.^24,25^ It holds great promise not only for unraveling complex crystallization processes observed in nature but also for enabling the synthesis of advanced functional materials, including crystalline soft and organic materials, with practical contributions to structural diversity in materials for nanoscience and nanotechnology.^26^ A key factor in guiding such crystallization pathways is the nature of intra- and intermolecular interactions among molecular building blocks.^27^ Therefore, the ability to predict and design amorphous intermediates by tailoring molecular interactions, for example, hydrogen bonding and short-range van der Waals forces, offers new avenues for constructing materials.^28^ For instance, linear dipolar molecules, through dipole–dipole interactions, can drive the formation of ordered anisotropic supramolecular structures such as one-dimensional (1D) nanowires, nanoribbons, and two-dimensional (2D) nanosheets.^29,30^ Molecules containing linear functional groups, like organic azides, further expand the design space by introducing stereochemical diversity and enabling directional control over molecular packing.^31^ Despite these advances, a mechanistic understanding of nonclassical crystallization in soft and organic materials remains limited. Compared to inorganic crystals, the formation mechanisms, interfacial structures, and dynamic behaviors of these systems are more challenging to investigate without perturbing their delicate nature, resulting in significant knowledge gaps.^27,32^

In this study, we observed that the amorphous–crystalline transformation assists spontaneous bending-to-straightening behavior in sugar azide 2,3,4,6-tetra-*O*-acetyl-β-d-glucopyranosyl azide (GluA), as shown in Scheme 1. This bending-to-straightening behavior evolves from bending to twisting and finally to the formation of a straight crystalline rectangular hollow tube through a three-step growth process that progresses from the amorphous phase over locally crystallized intermediates to the crystalline phase (Figure 1a). To elucidate the amorphous–crystalline transformation principles underlying the bending-to-straightening behavior, we combined molecular dynamics (MD) simulations of the amorphous phase, intermolecular energy calculations of the crystalline phase, and multiple time-resolved experimental techniques, including time-series optical microscopy, in situ Raman spectroscopy, and circular dichroism (CD) spectroscopy. Furthermore, systematic investigation of sugar azide stereoisomers (Scheme 1) of 2,3,4,6-tetra-*O*-acetyl-α-d-glucopyranosyl azide (GluAA), 2,3,4,6-tetra-*O*-acetyl-β-d-mannopyranosyl azide (ManA), and 2,3,4,6-tetra-*O*-acetyl-β-d-galactopyranosyl azide (GalA) reveals that the bending-to-straightening behavior is governed by a synergistic interplay between the azide group and sugar skeleton, enabling tunable growth directionality and structural dimensionality.

**Scheme 1:**
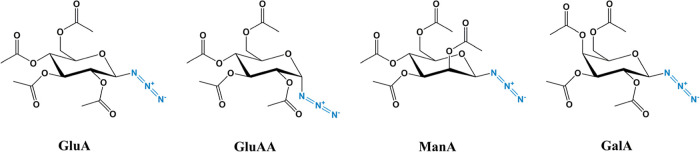
Chemical Structures of Azide-Functionalized Sugars Studied in This Work

**Figure 1:**
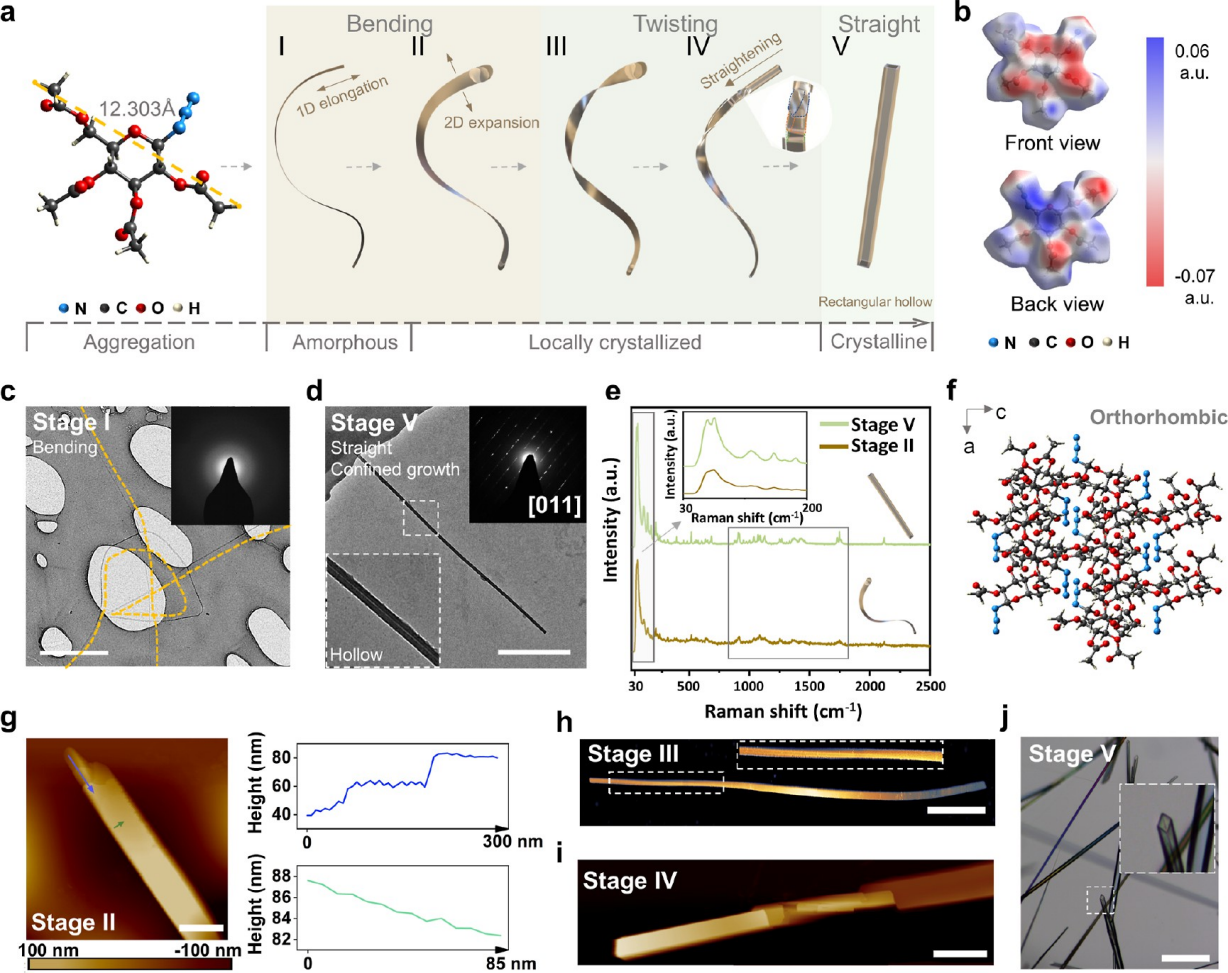
Amorphous–crystalline transformation enabling bending-to-straightening behavior with helical deformation. (a) Schematic illustration of the hierarchical structures from Stage I to V during bending-to-straightening behavior. The left image shows the chemical structure of GluA. The cracked structure of the helical deformation at Stage IV is highlighted by the blue, orange, and green dashed lines. (b) Electrostatic potential maps (in a.u.) of the GluA molecule. (c) Transmission electron microscopy (TEM) image of Stage I nanowire. The inset shows the SAED pattern of the nanowire. (d) TEM image of the rectangular hollow tube of Stage V obtained through confined evaporation (details refer to Methods). The inset shows the SAED pattern of the rectangular hollow tube. (e) Raman spectra of Stage II and Stage V. Significant differences are observed between Stage II and Stage V in both the mid- and low-frequency spectral regions (gray frame). (f) Crystal packing of GluA in Stage V. (g) Atomic force microscopy (AFM) image shows the layered stacking features of Stage II. The line profile plots the height trace of the correspondingly colored arrow. (h) AFM image of helical deformation in Stage III. (i) AFM image of straightening starting end and rupture of helical deformation of Stage IV. (j) Optical microscopy images of the straight crystalline rectangular hollow tube in Stage V. Scale bars: (c) 1 μm, (d) 1 μm, (g) 270 nm, (h) 3 μm, (i) 3 μm, and (j) 100 μm.

## Experimental Section

### Materials

d-glucose, acetic anhydride, iodine, sodium thiosulfate, N, N′-dimethylformamide (DMF), and methanol (MeOH) were purchased from Sigma-Aldrich (Germany). Hydrogen bromide, sodium carbonate, sodium azide, dichloromethane, sodium sulfate, acetic acid (AcOH), and ethyl acetate (EtOAc) were purchased from TH Geyer (Germany). 2,3,4,6-Tetra-*O*-acetyl-β-d-glucopyranosyl-isothiocyanate (GluI) was purchased from TCL Europe. GluAA and ManA were purchased from Biosynth Ltd. (UK). Unless otherwise specified, the chemicals were used without further purification and were of analytical grade or higher. Deionized water (DI water) was used throughout the experiments.

Synthesis pathways of GluA and GalA are shown in Supporting Information.

### Preparation of the Straight Crystalline Rectangular Hollow Tube in Stage V

The GluA powder (16 mg) was dissolved in a mixed MeOH (1 mL) and H_2_O (1 mL) solution in a glass vial. The glass vial was sealed and placed in the 4 °C refrigerator for 2 days after heating to 353 K for 5 min to form the homogeneous solution. Straight crystalline rectangular hollow tubes were harvested at the bottom of the glass vial.

A confined-growth straight crystalline rectangular hollow tube for TEM observation was prepared by dropping a 5 μL aliquot of a 1 mg/mL GluA solution (in 1:1 MeOH/H_2_O) onto an ultrathin carbon-film-coated Au grid. The grid was then covered with a Petri dish to slow evaporation.

### Preparation of the Intermediate Samples of Stages I–IV

Intermediate samples representing Stages I–IV were obtained by terminating the self-assembly process correspondingly. This was achieved through the controlled evaporation of the GluA solution (at a concentration of 1 mg/mL in a MeOH/H_2_O mixture), with specific intermediate stages acquired by adjusting the evaporation time. Obtained samples were employed to perform Raman spectra analysis and morphology analysis by using AFM, scanning electron microscopy (SEM), and TEM.

### In Situ Observation of the Bending-to-Straightening Behavior

The GluA solution (8 mg/mL in a 1:1 MeOH/H_2_O mixture) was sealed in a cuvette with a thickness of 1 mm, allowed to undergo a temperature decrease from 353.15 to 277.15 K, and aged at 277.15 K. The prepared sample was employed for optical microscopic observation and interval CD measurements.

The in situ observations of the bending-to-straightening behavior using GluAA, ManA, and GalA were performed identically to those for GluA.

### Characterization

The optical images and in situ observation of samples were recorded using an optical microscope, an Olympus BX-51, and polarized images of samples were collected using an Eclipse 600 microscope from Nikon. The polarized Raman spectra were recorded via a LabRAM HR Evolution (HORIBA France SAS) system. An LEO supra-35 high-resolution field emission scanning electron microscope (Carl Zeiss AG, Germany) was used to characterize the microstructure of various samples, and the targeted voltage was 5 kV. Prior to SEM measurements, a thin layer of gold nanoparticles was coated on the sample surface. Atomic force microscopy (AFM) characterization was performed in the dry state by using a Multimode 8 AFM (Bruker, Karlsruhe, Germany) with a NanoScope V controller in an ambient environment. X-ray diffraction (XRD) patterns were recorded at ambient temperature using a Bruker D8 Discovery (X-ray wavelength = 1.54  Å, Cu Kα radiation). The transmission electron microscopy (TEM) observations were conducted using a CM 12 (Philips, the Netherlands), and selected area electron diffraction (SAED) was acquired using an FEI Tecnai transmission electron microscope operating at 120 kV. Circular Dichroism (CD) spectra were obtained using a JASCO J-1700 CD spectrometer (JASCO Corporation, Japan). The SCXRD data were collected by using Mo Kα radiation and a Bruker Photon III detector. The *G*-value was calculated by spectra analysis (JwStdAnalysis) in the meantime. The BJDF prediction was done by Material Studio 2020. Cryo-EM was carried out with a Titan Krios G4 (Thermo Fisher Scientific) operating at 300 kV.

## Results and Discussion

A spontaneous bending-to-straightening behavior is observed during the cooling process in a methanol–water mixture, starting from a bending amorphous nanowire (Stage I), growing into a locally crystallized nanoribbon with helical deformation (Stages II–IV), and finally forming a stable phase of a straight crystalline rectangular hollow tube (Stage V), as shown in Figure 1a. The transition involves an upscaling from a GluA molecule with the diameter of 12.303 Å to an axial length reaching up to 1.3 cm by 0.1 mm^2^ straight crystalline rectangular hollow tube (Figure S1). The glucopyranose ring’s hydroxyl groups at positions 2, 3, 4, and 6 are acetylated, with an azide group attached at the anomeric carbon, forming a linear structure. The charge distribution of GluA is visually depicted by electrostatic potential (ESP) maps (Figure 1b). The collinear dipolar arrangement of the azide group, where the three nitrogen atoms are aligned along a straight line, is illustrated by the electrostatic potential distribution, with a positive potential (shown in blue) concentrated on the central nitrogen and negative potentials (shown in red) localized on the terminal nitrogens. The spatial configuration of the sugar skeleton directs this negative potential to one side of the pyran ring, while the opposite side shows a more positive potential. This distribution supports the self-assembly process, initiating GluA molecule aggregation (Figure S2).

Crystallographic characteristics of Stage I and Stage V were analyzed by using TEM-selected area electron diffraction (SAED), Raman spectroscopy, and single-crystal X-ray diffraction (SCXRD). As shown in Figure 1c, a bending nanowire was captured and exhibited an amorphous nature as verified by TEM-SAED. Due to the size limitations of Stage V, confined growth was employed to obtain a scaled-down straight crystalline rectangular hollow tube, and its single-crystal nature was verified by TEM-SAED, interpreted by typical single-crystal strips with regular gap distance that are indexed to the [011] zone axis with *d* (*hkl*) = 1.078 nm (Figure 1d). Raman spectra of crystalline and amorphous solids with identical composition differ significantly due to the presence or absence of spatial order and long-range translational symmetry.^33^ Raman spectral analysis was performed on characteristic samples from Stage II and Stage V (Figure S3). The Raman spectrum of the Stage II samples displays broader, less distinct bands compared to that of Stage V, particularly in the low-frequency and mid-frequency regions, indicating the amorphous nature of Stage II and the crystalline structure of the Stage V sample (Figure 1e).^34^ We measured the crystallographic structure of GluA, which agrees with the reported data.^35^ The straight crystalline rectangular hollow tube in Stage V reveals an orthorhombic packing diagram with a *P*2_1_2_1_2_1_ chiral space group (Figures 1f and S4 and Table S1). The packing patterns of the *ac* plane exhibit a spatial orientation of the linear azide group (N_3_), as shown in Figure S5. The angle between N_3_ and the *a*-axis is approximately 22.9°, with dihedral angles of 47.3° between (011) and (010) and 42.7° between (001) and (010). The packing exhibits a sandwich herringbone pattern with a segment (blue square) distance of 15.87 Å.

The initial radius expansion of the bending nanowire occurs unevenly. Subsequently, the nanowire in Stage I evolves into a bending nanoribbon structure in Stage II (Figure S6). The nanoribbon exhibits graded 2D layer stacking, as illustrated in Figure 1g. At the growth end, the stacking layers show larger steps, approximately 20 nm in height, as highlighted in the blue line profile. In contrast, the surface of the nanoribbon displays layer stacking with a depth of around 1 nm, as indicated by the green line profile. As growth proceeds, the step edges at the growing end display a different progression. Certain regions advance at a faster rate than others, thereby resulting in twisting motion driven by the growth asymmetry, as shown in Figure S7. Consequently, helical deformation emerges and evolves into two distinct structural modes, as illustrated in Figure 1a (Stage III and Stage IV). The twist is uniaxial with a continuous twist surface at Stage III (Figures 1h and S8). The propagating growth involving radial expansion led to a transition in the twisted morphology, which ultimately fractured into cracked, plate-like crystallites due to local crystallization (Figures 1i and S9). At the same time, the starting end exhibits a straightening behavior, characterized by thickening in the out-of-plane direction and narrowing in the in-plane direction perpendicular to the longitudinal axis, in contrast to the stacking end (Figure S10). This loss of flexibility of the starting end indicates the occurrence of the crystallization process. Ultimately, the crystalline structure with a rectangular hollow was formed (Figures 1j and S11).

The bending-to-straightening behavior was analyzed through a series of in situ observations. The helical spinning motion from the starting end view of the nanoribbon was recorded, as shown in Figure 2a and Movie 1. With spinning, twist knots flash from the stacking to the starting end, traced by yellow arrows. Framed in white/red, twisting structures and crystalline growth (blue) are highlighted. By confined sample preparation, an independent twist was captured by SEM observation, clearly showing the helicity of the knot (Figure S12). In situ observation of the stacking end view examined bending-to-straightening behavior (Figure 2a, Movie 2). The nanoribbon’s bending motion was restrained by crystallization, gradually leading to straightening properties (blue dashed line). Eventually, it formed a straight crystalline rectangular hollow tube with dark outlines and lighter inner regions, as shown with the optical microscope image. Bending-to-straightening behavior can also be executed in locally crystallized areas, as shown in Figure 2b and Movie 3. The crystalline regions are marked by straight sections within the white frame, while the curved blue lines address the bending deformation zones. The orange frames capture the spinning motion, which spans approximately 540°. As crystallization progressed, the bending-to-straightening behavior was completed. Similar phenomena of bending-to-straightening behavior facilitated by local crystallization were observed, as shown in Movie 4. Due to technical limitations, in situ observations were limited to the onset of the bending-to-straightening behavior and the crystalline growth of the rectangular hollow tube. The longitudinal growth rate during the bending-to-straightening behavior from Stage III to Stage IV displays a large error range with a decay. In contrast, subsequent growth of the straight crystalline rectangular hollow tube exhibits a constant growth rate of around 29.97 μm/s (Figure S13). Furthermore, the growth exhibits pronounced anisotropy with the radial expansion rate being approximately 63 times slower than the longitudinal growth rate. Cryo-EM was employed to investigate the local crystallization phenomenon (Figure 2c). Specifically, a nanoribbon at Stage II was examined by using SAED along the longitudinal axis. Due to the sample’s thickness and beam-sensitive organic molecular crystalline nature, obtaining a high-quality diffraction pattern was challenging. Nevertheless, the diffraction signals were inconsistently observed across the six sampled positions, indicating the presence of both amorphous and crystalline regions in Stage II.^36^

**Figure 2:**
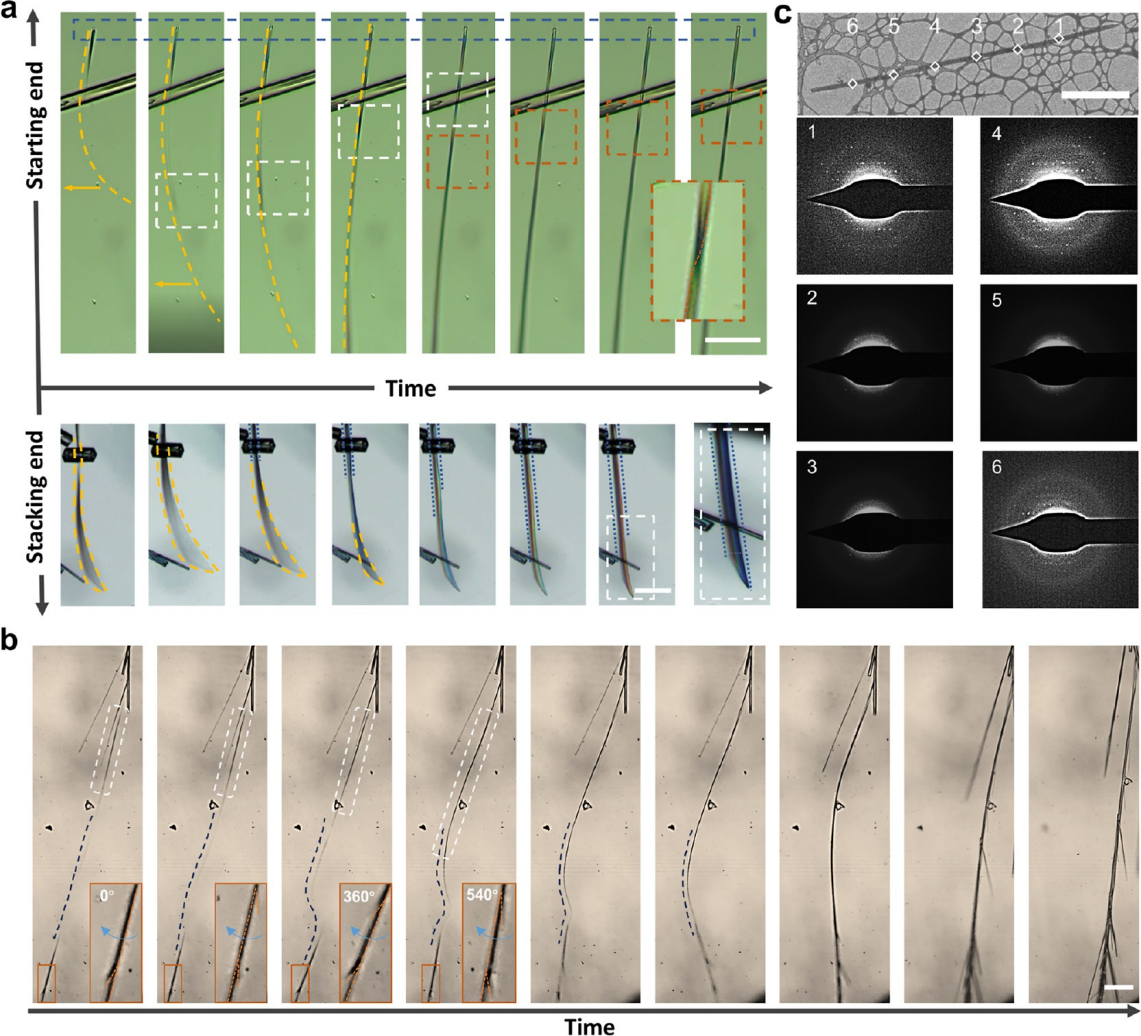
In situ observation of the spontaneous bending-to-straightening behavior. (a) Optical microscope observations of the bending-to-straightening behavior along the growth trajectory from the starting end to the stacking end (from left to right). (b) Optical microscope observations of the bending-to-straightening behavior during local crystallization. White frames highlight the extension of the crystalline area, while blue dashed lines address the bending deformation zones. The zoomed-in images in each panel are highlighted with corresponding orange frames. The straight crystalline structures adjacent to the targeted sample in (a) and (b) share the same crystallographic structure but have already completed the bending-to-straightening process. (c) Cryo-EM image of the Stage II sample with corresponding SAED images of the selected areas. Scale bar: (a) 50 μm, (b) 100 μm, and (c) 5 μm.

Polarized Raman spectroscopy was employed to determine the anisotropic structures during the morphological transition.^37^ Raman spectra of GluA, penta-*O*-acetyl-β-d-glucopyranose (Glu-OAc), and glucose were recorded (Figure S14). The setup for the angular-dependent Raman spectroscopy is shown in Figure S15. The azide group (N_3_), as the linear dipolar structure in the GluA molecule, aids in analyzing the orientation differences between the twist in Stage III and the straight crystalline rectangular hollow tube in Stage V.^38^ The resultant angle-resolved Raman intensities for the two structures are shown in Figure S16. Polar plots are prepared to visualize the differences of the molecular orientation between the two structures (Figure 3a,b). The flawless spindle-shaped polar diagrams (20°–40°) highlight the anisotropy and highly oriented crystal packing of the straight crystalline rectangular hollow tube in Stage V, while the twist structure differs. The polar diagrams corresponding to the regions after and before the twist exhibit approximate mirror-symmetric patterns, with a symmetric axis near 350° that shows rough alignment with the longitudinal axis. Notably, after the twist, the axial orientation of the polar plot aligns with that of the straight crystalline rectangular hollow tube, collectively confirming the presence of a uniaxial twist structure. Moreover, the irregular polar plot patterns of the twisted structure reveal a multipopulation distribution of orientations beyond the primary axial alignment, suggesting the presence of misaligned crystallites formed during local crystallization.

**Figure 3:**
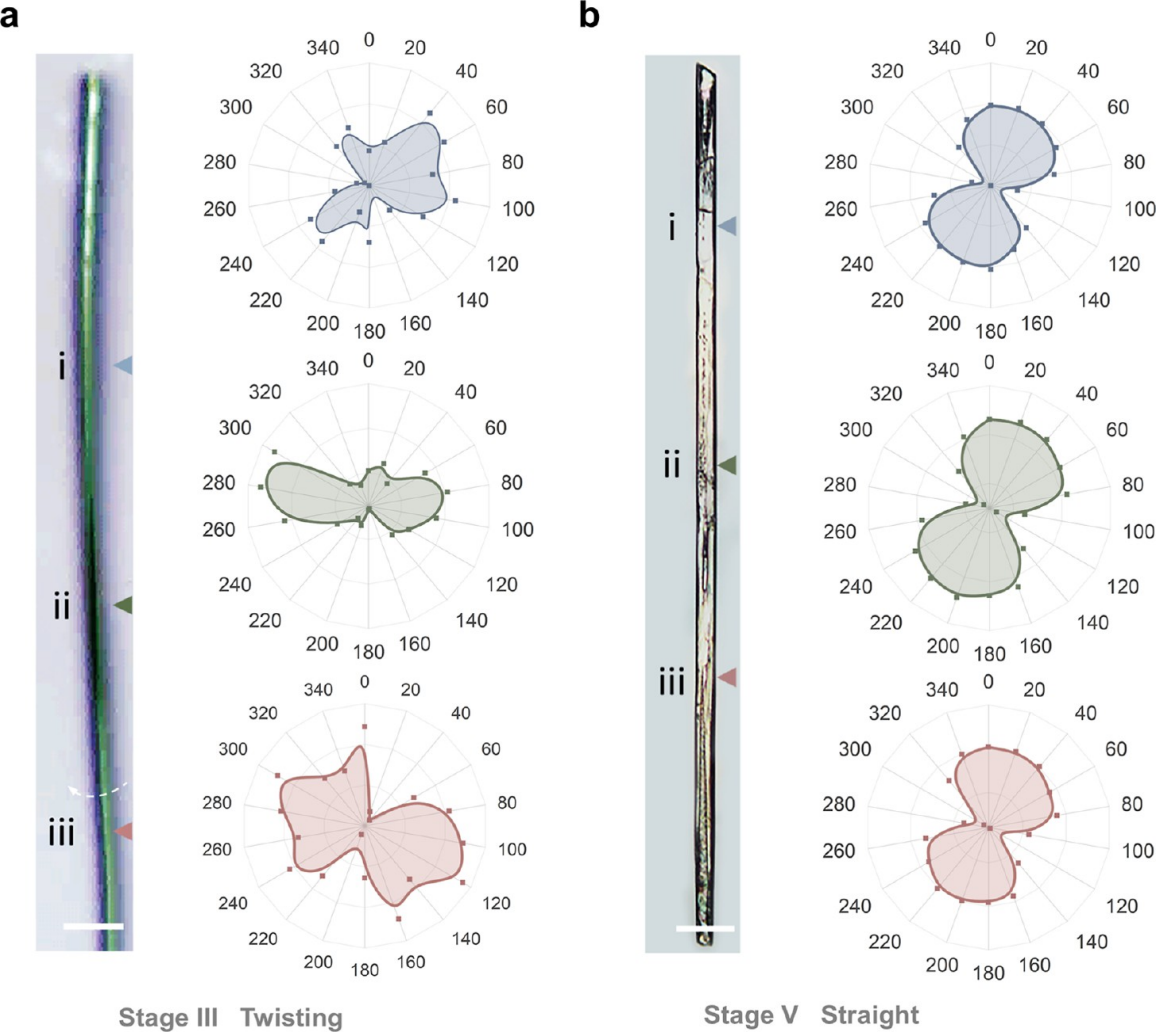
Angular-dependent Raman spectroscopy analysis of the hierarchical structures during bending-to-straightening behavior. (a) Orientation analysis of the twist in Stage III, based on polar plots of angular-dependent Raman spectra measured at selected points: (i) after the twist, (ii) on the twist, and (iii) before the twist. (b) Orientation analysis of the straight crystalline rectangular hollow tube in Stage V, based on the polar plots of the angular-dependent Raman spectra acquisition measured at specific locations on the object. The polar plots in (a) and (b) refer to the normalized intensity of asymmetric stretching vibrations of the azide group (*v*_as_(N_3_)) at 2117 cm^–1^ relative to the asymmetric stretching vibrations of the CH_3_ group (*v*_as_(CH_3_)) of the acetyl group at 2942 cm^–1^. The polar plots are plotted against the polarization azimuthal angles (θ) of the incident laser. Here, θ is defined as 0° when the polarization direction of the incident light is parallel to the elongated direction of the object.

Mechanical strain analysis using Raman spectroscopy was applied to the straightening intermediate in Stage IV and the straight crystalline rectangular hollow tube in Stage V to compare the structure evolution of the bending-to-straightening behavior.^39^ The intermediate structure displays a straightening behavior at the starting end while maintaining bending behavior at the stacking end (Figure 4a). Fourteen discrete locations revealed a progressive Raman shift gradient. The starting end (spot 1) shows a Raman shift higher than that of the stacking end (spot 14), suggesting localized compressive strain induced by crystallization-assisted bending-straightening behavior (Figure 4b). In contrast, the straight crystalline rectangular hollow tube exhibits a uniformly higher Raman shift than the intermediate structure in Stage IV. This spectroscopic evidence suggests the higher compressive strain with stronger intermolecular interaction exists in the straight crystalline rectangular hollow tube in Stage V (Figure 4c,d). Raman shifts of three equidistant spots on the bending nanoribbon in Stage II were considered (Figure 4e). The outer bend shows a lower Raman shift, characteristic of tensile strain leading to looser molecular packing, while the inner bend exhibits a higher Raman shift, suggesting compressive strain caused by the deformation of bending (Figure 4f).^40^

**Figure 4:**
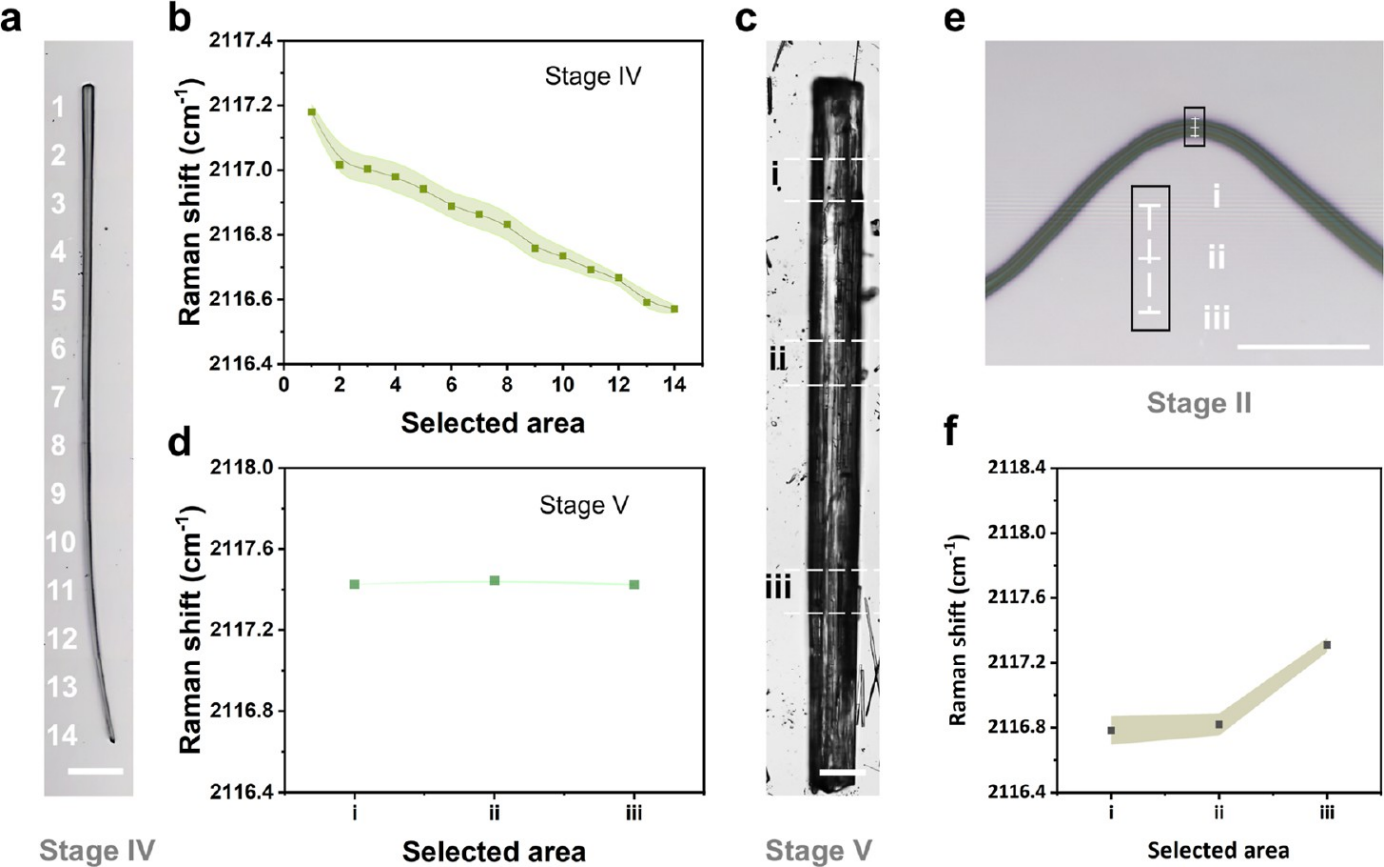
Raman spectroscopy of mechanical strain analysis. (a) Optical microscope image of straightening intermediate in Stage IV. (b) Strain dependence of Raman shifts of the *v*_as_(N_3_) of the selected positions in (a). (c) Optical microscope image of the straight crystalline rectangular hollow tube in Stage V. (d) Strain dependence of Raman shifts of the *v*_as_(N_3_) of the selected positions in (c). (e) Optical microscope image of the bending nanoribbon in Stage II with the selected positions: (i) outer bend, (ii) middle, and (iii) inner bend. (f) Strain dependence of Raman shifts of the *v*_as_(N_3_) of the bending nanoribbon in (e). The shaded regions in (b), (d), and (f) represent the error bars for the measured points. Scale bar: (a) 250 μm, (c) 100 μm, and (e) 20 μm.

The in situ chiroptical properties of the self-assembly of GluA molecules into straight crystalline rectangular hollow tubes were studied using circular dichroism (CD) spectroscopy. The intrinsic GluA molecule exhibits a positive CD signal around 268 nm, indicating left-handed molecular chirality (Figure S17).^41^ In comparison, the straight crystalline rectangular hollow tube in Stage V lacks a CD signal. Temperature–time CD measurements showed that the CD signal shape of GluA in solution remained unchanged during cooling (Figure S18). After 2711 s of aging at 277.15 K, a red shift of the peak to approximately 276 nm occurred (yellow dashed line, Figure 5a), while the signal intensity at 268 nm gradually decreased. The chirality diminishes as it transitions into a CD-silent straight crystalline rectangular hollow tube. To evaluate the chiroptical activity, the asymmetry factor *g* (*g*_CD_) was calculated from CD and absorption spectra, shown in Figure 5b. The local maximum *g*_CD_ value is 0.012, which decreases after 1645 s. Alongside CD spectra, linear dichroism (LD) spectra exhibit a signal appearing after 1654 s and gradually increasing thereafter. It demonstrates anisotropic growth of GluA during the self-assembly process from Stage I to Stage V (Figure 5c).

**Figure 5:**
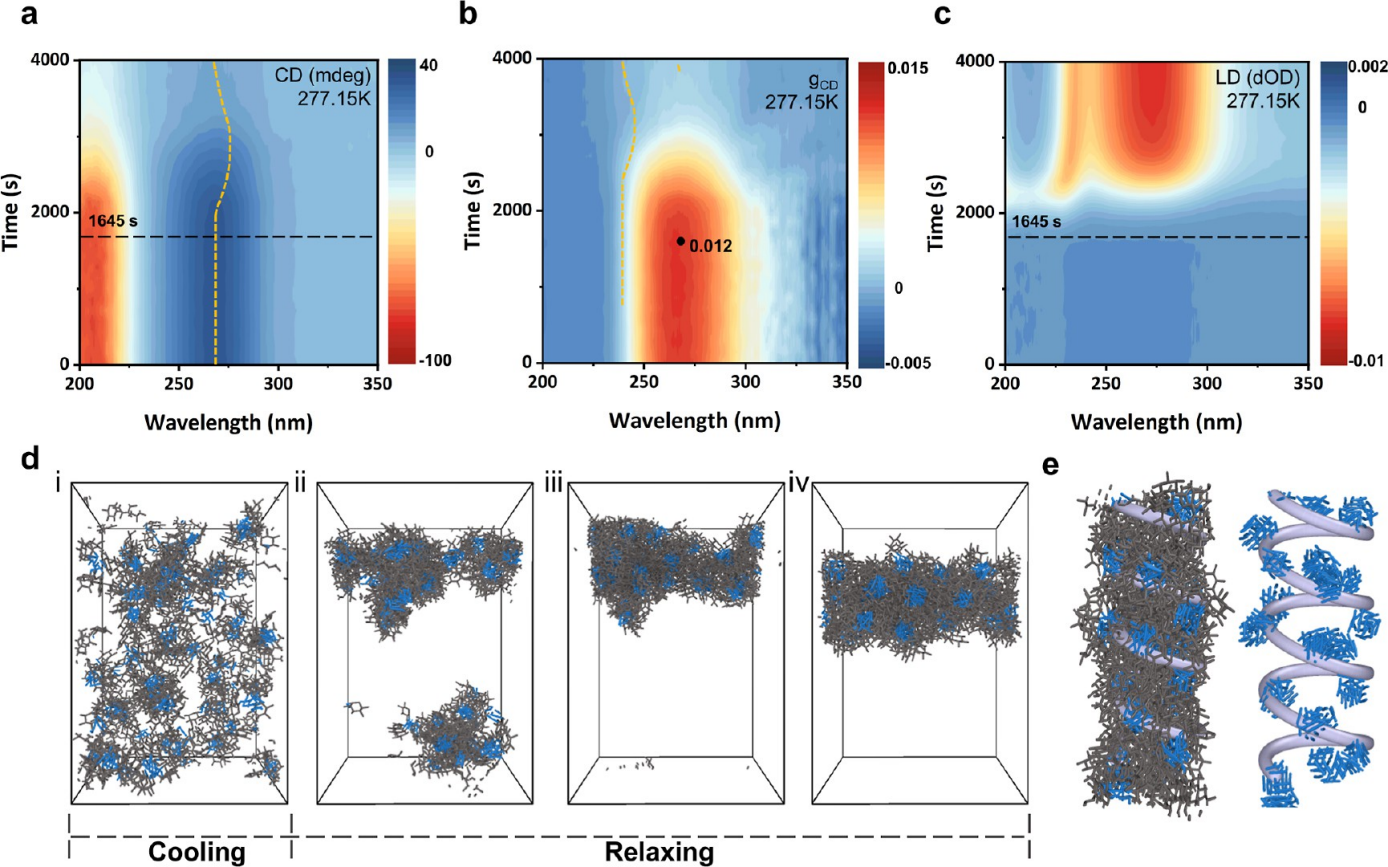
Anisotropic self-assembly and the MD simulation in the formation of the amorphous nanowire. (a) Interval CD spectra of GluA in methanol–water solution under 277.15 K aging for 4000 s, and the cuvette path length is 1  mm. (b) Asymmetry factor *g*_CD_ calculated from the CD spectra. (c) LD spectra of GluA in methanol–water solution under 277.15 K aging for 4000 s, and the cuvette path length is 1  mm. The temperature–time interval CD measurements capture the chiroptical changes during the self-assembly of GluA molecules into straight crystalline rectangular hollow tubes, which involves an amorphous–crystalline transformation that induces helical deformation and enables the bending-to-straightening behavior. The measurements begin with a GluA solution and conclude with a suspension of straight crystalline rectangular hollow tubes. (d) Formation kinetics of the amorphous anisotropic aggregates in Stage I: (i) the original state of molecular solution, (ii) small clusters formed by molecules after cooling for 1000 τ and relaxing for 4000 τ, (iii) one cluster formed after the relaxation of 48,000 τ, and (iv) the amorphous anisotropic structure formed by molecules after the relaxation of 148,000 τ. The coarse-grained nitrogen and carbon beads were represented using blue and gray sticks, respectively. (e) The helix structure of Stage I at reduced temperature 1.4 (for more details, see Note 1). The purple spiral line represents the helical structure of the aggregation. The right side is the helix structure of aggregated azide group segments along the spiral line.

To delve into the formation kinetics of anisotropic amorphous nanowires in Stage I, a coarse-grained model of the GluA molecules was employed and run by MD simulations (Note 1). The growth of the amorphous structure was simulated as shown in Figure 5d. GluA molecules were initially randomly dispersed (Figure 5di) and then gradually self-assembled into anisotropic cylinders (Figure 5dii–iv). Due to strong attraction between nitrogen beads (linear azide group), small clusters formed after 4000 τ of relaxation (Figure 5dii), which then merged into a larger cluster by 4800 τ (Figure 5diii). After 148,000 τ, the cluster evolved into a well-defined anisotropic structure (Figure 5div). Here, τ denotes the MD simulation time unit. The experimental aggregations not only displayed anisotropy but also exhibited a helical structure. As shown in Figure 5e, the nitrogen beads formed a cylindrical helix, which can be captured by the following function:

1 $$\{\begin{array}{l} x=a\;\cos\theta \;\\ y=a\;\sin\theta \\ z=b\theta +c\sin\theta \end{array}$$

where *a* is the radius of the helix, *b* is the diameter, which represents the distance growth per θ, and *c* sin θ is used to shift the curve at the *y*-axis. The coefficients were chosen as *a* = 7.5 σ, *b* = 1.58 σ, and *c* = 2.2 σ. Here, σ corresponds to a coarse-grained molecule bead and is on the order of σ = 0.1 nm. The simulation results revealed the formation of a helical structure at amorphous Stage I, consistent with the red shift observed in the time-dependent CD spectra.

Intermolecular interaction analysis was employed to investigate crystalline molecular packing evolution during the amorphous–crystalline transformation. A cluster of molecules was generated based on SCXRD by including all atoms within a 3.8 Å radius of the central molecule, ensuring a complete fragment around the molecular centroid for intermolecular interaction analysis. Intermolecular interaction energies were calculated using the monomer electron density derived from B3LYP/6-31G­(d,p) to evaluate the electrostatic, polarization, dispersion, and exchange-repulsion components between GluA molecules,^42^ as shown in Figure S19. Among these interactions, the dispersion energy, representing van der Waals forces, plays a dominant role. In addition, the strongest total intermolecular interaction energy of −42.2 kJ/mol observed in a dimeric pair is a key driving force for crystallization, leading to the formation of a sandwich herringbone configuration (Figure 6a). The second strongest interaction, −35.0 kJ/mol, promotes rapid growth along the *a*-axis, where the linear dipolar azide groups are arranged collinearly over a long-range (Figure 6b). The Bravais–Friedel–Donnay–Harker (BFDH) crystal morphology model further confirmed that the *a*-axis of the crystallographic coordinate system corresponds to the longitudinal axis of the sample coordinate system (Figure S20). Two pairs with energies of −27.6 and −21.6 kJ/mol indicate the orientation of the (011) lattice (Figure 6c). The asymmetry in this repeating unit arises from both intermolecular energies and crystal packing. As shown in Figure 6d, the lower intermolecular energy of −5.0 kJ/mol is asymmetrically positioned, making it easier to break or alter weaker interactions.

**Figure 6:**
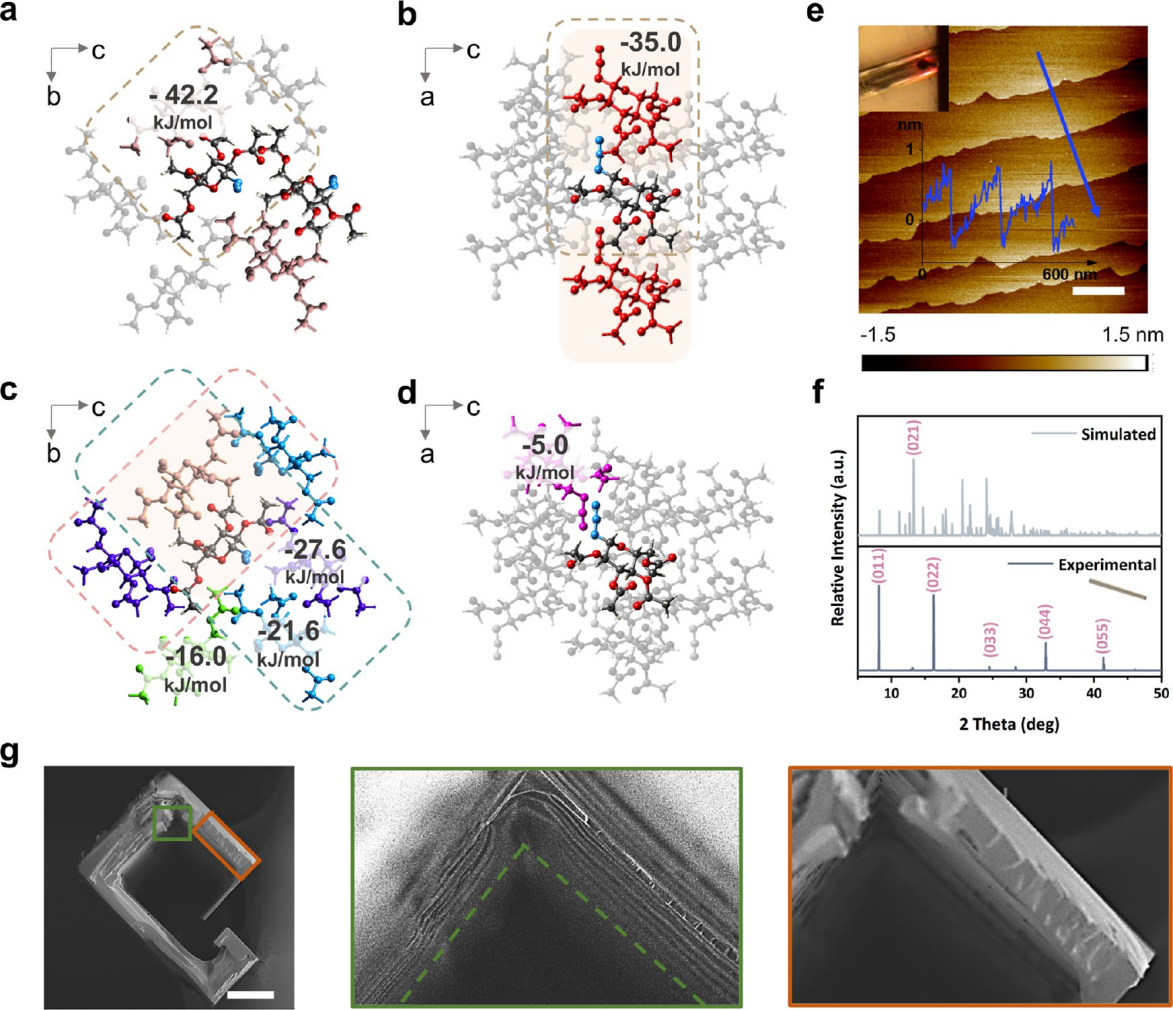
Intermolecular energy calculation of the straight crystalline rectangular hollow tube. (a–d) A cluster of molecules was generated based on SCXRD by including all atoms within a 3.8 Å radius of the central molecule, ensuring a complete fragment around the molecular centroid for intermolecular interaction analysis. (a) The total energies surrounding a single GluA molecule, positioned within the dimeric pair in the sandwich herringbone molecular packing. (b) The total energies around a single GluA molecule along the *a*-axis. (c) The total energies of surrounding molecules around a single GluA molecule. (d) The asymmetrical intermolecular interaction around a single GluA molecule along the *a*-axis. (e) AFM image of surface analysis for the straight crystalline rectangular hollow tube in Stage V. The inset shows the optical microscope image of the measured area and the line profile plotted along the blue arrow. (f) Powder X-ray diffraction (PXRD) analysis was performed on the straight crystalline rectangular hollow tube in Stage V, and the resulting pattern was compared with the simulated PXRD pattern calculated from the GluA SCXRD data. Measurements were conducted with the aligned crystal oriented parallel to the X-ray beam. (g) SEM images showing dislocations in the straight crystalline rectangular hollow tube at Stage V. The zoomed-in images on the right correspond to the colored frames and dislocation lines marked in the cross-sectional image. Scale bar: (e) 200 nm and (g) 50 μm.

The Hirshfeld surface gives a unique signature of a molecule in a crystal depending on the surrounding.^43^ The Hirshfeld surface of GluA is shown as contact zones shorter than van der Waals radii that are marked as red areas (Figure S21). 2D fingerprint scatterplots of *d*_e_ and *d*_i_ uniquely identify each type of interaction in the crystal structure. In the case of GluA, the most dominant contacts are H···H contacts, constituting the highest fraction of 37%, which is followed by those of O···H type contacts, contributing to 35%. Other close contacts are also present, which include H···N contacts (18.8%), weak C···H interactions (1.1%), C···O interactions (2.3%), N···O interactions (3.6%), and O···O interactions (2.2%). These results suggest the dominance of van der Waals interactions and hydrogen bonds. The six H-bond networks in the generated molecular cluster were analyzed (Figure S22 and Table S2). As nonclassical C–H···O interactions are distinct from van der Waals interactions and often display directionality, they are indicative of underlying orbital contributions.^44^ Three H-bonds within the building block dimers stabilize the sandwich herringbone packing (Figure S23). Additionally, the H-bonds between the molecules along the *a*-axis (Figure S24) synergistically enhance the preferential stacking of the molecules along the *a*-axis. The other two types of H-bonds facilitate the stacking of GluA molecules along the *b*-axis and *c*-axis, respectively, as shown in Figure S25. Particularly, the length of the H-bond along the *c*-axis is 2.295 Å, which is stronger than the H-bond of 2.493 Å along the *b*-axis. Therefore, the van der Waals interactions and the nonclassical C–H···O interactions together contribute to the crystallization process during the amorphous–crystalline transformation.

In addition to the proposed sandwich herringbone molecular packing mode, a layered stacking structure was also observed. AFM revealed stacking features with a thickness of approximately 1 nm on the outer surface of the straight crystalline rectangular hollow tube in Stage V (Figure 6e). PXRD analysis of aligned straight crystalline rectangular hollow tubes with diffraction peaks at (011), (022), (033), (044), and (055) indicating layered stacking along {011} faces (Figure 6f). Furthermore, the experimental PXRD pattern exhibits an obvious preferred orientation of texture along the [011] direction, notably differing from the simulated signal where the (021) peak is the most intensive while the {011} reflections are weak. The results are further validated by comparison with the rotated sample and the GluA powder sample (Figure S26). While it is undeniable that layer stacking and expansion will also promote growth in all crystallographic dimensions, the layer-by-layer stacking mechanism of self-assembly is clearly adapted here.^45^ In addition, we observed dislocations during crystal growth, as shown in Figure 6g. The cross-sectional view reveals that dislocation lines are oriented in two directions, along the longitudinal axis (green frame) and the radial direction (orange frame). This indicates the facilitation of screw dislocation toward the longitudinal growth of the hollow crystal.^46^ Meanwhile, edge dislocation also contributes to radial distortion and influences the formation of the unclosed rectangular hollow.^47^ Hence, we propose the initiation of helical deformation at Stage III is driven by the interplay of this shear stress arising from the screw dislocations and the concurrent stress relaxation, which is a consequence of the molecular packing rearrangement during the amorphous–crystalline transformation.^48,49^ Notably, the intrinsic chirality of the GluA molecule dictates the observed left-handedness of the helical deformation in both Stage III and Stage IV, facilitated by the role of screw dislocations.^50^

The stereostructure of GluA governs the amorphous–crystalline transition that drives the bending-to-straightening behavior. To explore the universality of the bending-to-straightening behavior in sugar azides and to underscore the importance of stereochemical configuration, three more stereoisomers, GluAA, ManA, and GalA, were chosen for investigation (Figure 7a). Furthermore, all of these molecules exhibit the bending-to-straightening behavior during the self-assembly process. GluAA with the linear dipolar azide group at the anomeric carbon in α (downward) configuration, different than GluA in the β (upward) configuration, also showed a bending-to-straightening behavior. Notably, this motion exhibited the same behavior as that shown by GluA, particularly in the preferential growth direction along the longitudinal axis, resulting in straight crystals with a high aspect ratio (Figure 7b). In contrast, when the azide group was replaced with a different linear dipole structure, specifically the isothiocyanate group in 2,3,4,6-tetra-*O*-acetyl-β-d-glucopyranosyl isothiocyanate (GluI) at the anomeric carbon, only anisotropic crystals were formed. Under these conditions, the change from an amorphous to a crystalline state, which previously caused the bending-to-straightening behavior, did not occur (Figure S27). Moreover, the absence of a linear dipolar structure (Glu-OAc) resulted in no observable anisotropic crystalline structure (Figure S28). These findings indicate that the azide group is a key factor in achieving the bending-to-straightening behavior.

**Figure 7:**
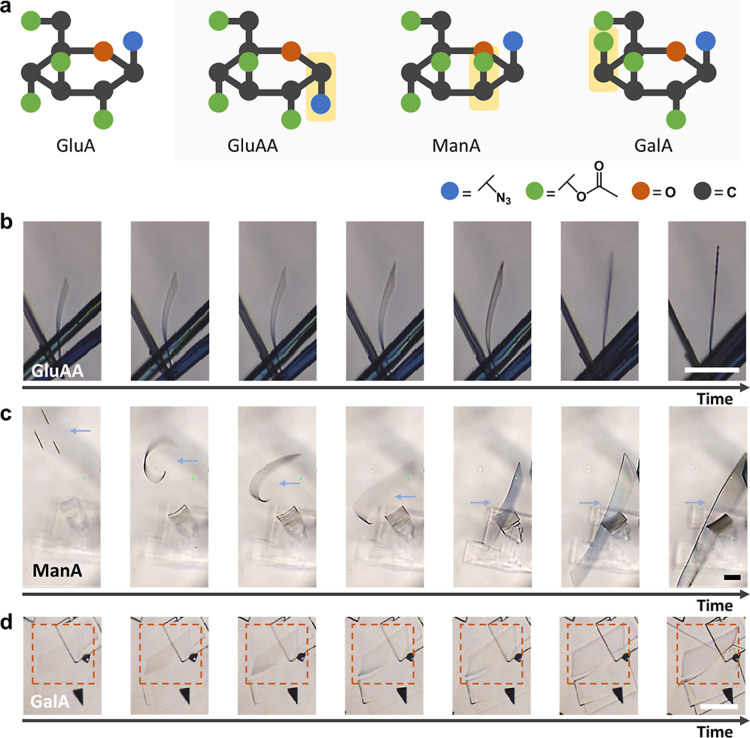
Investigation of the bending-to-straightening behavior using various sugar azide stereoisomers. (a) Stereochemical configurations of sugar azide stereoisomers: 2,3,4,6-tetra-*O*-acetyl-α-d-glucopyranosyl azide (GluAA), 2,3,4,6-tetra-*O*-acetyl-β-d-mannopyranosyl azide (ManA), and 2,3,4,6-tetra-*O*-acetyl-β-d-galactopyranosyl azide (GalA). Stereochemical differences relative to GluA are highlighted in yellow. (b–d) In situ observation of the bending-to-straightening behavior based on GluAA, ManA, and GalA. Scale bar: (b–d) 100 μm.

The bending-to-straightening behavior can be adjusted by altering the stereostructure of the sugar skeleton. When the acetyl group at C2 is axial (ManA) instead of equatorial (GluA), the width of the resulting bending nanoribbon is relatively larger than that formed from GluA (Figure 7c). Furthermore, if the acetyl group at C4 is axial (GalA), then the width of the assembled structure is even greater, resulting in a restriction of the bending motion (Figure 7d). This restriction is attributed to the limited vertical (out-of-plane) space available within a 1 mm cuvette. Due to the enhanced growth in the lateral in-plane direction (perpendicular to the longitudinal axis), these two molecules did not form a hollow structure but yielded 2D sheets instead. Therefore, the sugar ring stereostructure and the azide group synergistically govern the amorphous–crystalline transformation and dictate the directionality and dimensionality of growth during the bending-to-straightening behavior.

## Conclusion

This work elucidates the molecular and crystallographic origins of the spontaneous bending-to-straightening behavior using GluA molecules. The transformation proceeds hierarchically, beginning with bending amorphous nanowires, progressing through locally crystallized nanoribbons with helical deformation and culminating in straight crystalline hollow tubes mediated by screw dislocation. This structural evolution is directed by the combined influence of screw dislocation-induced shear stress, stress relaxation associated with the amorphous-to-crystalline transformation, and the intrinsic chirality of the GluA molecule. Crucially, the stereochemical configuration of the pyranose ring and the collinear dipole arrangement of the azide group act in concert to regulate the growth directionality and dimensionality. By establishing the interplay between molecular stereostructure and dipolar functional groups, this study advances the broader understanding of nonclassical crystallization pathways associated with mechanically responsive crystal growth and provides a framework for exploiting sugar azides as building blocks for adaptive, self-actuating materials.

## Supplementary Material











## Data Availability

The data supporting the plots in this paper and other findings of this study are available from the corresponding authors upon request.

## Notes

Methods; synthesis pathways; image of the straight crystalline rectangular hollow tube; TEM image and MD simulation; schematic of the polarized Raman spectroscopy setup; image of a bulk orthorhombic crystal of GluA; molecular packing images; SEM images; AFM images; cut profiles; growth rate plots; chemical structure and Raman spectra; schematic of the angular-dependent polarized Raman spectroscopy setup; angle-resolved Raman spectra; CD spectra and setup schematic; absorbance spectrum and temperature interval CD spectrum; intermolecular energy data; crystal morphology; Hirshfeld surface image and 2D fingerprint plot; PXRD patterns; chemical structures and OM images; crystallographic parameters; hydrogen bond information; structure and coarse-grained model and MD simulations of the GluA molecules; simulation details; details on the influence of bead concentration, temperature, and cooling rate; ^1^H NMR and ^13^C NMR spectra; and ESI-HRMS analysis results (PDF)
Movie 1: Bending-to-straightening behavior on the starting end (scale bar: 100 μm) (MP4)
Movie 2: Bending-to-straightening behavior on the stacking end (scale bar: 100 μm) (MP4)
Movie 3: Bending-to-straightening behavior among local crystallization areas 1 (scale bar: 100 μm) (MP4)
Movie 4: Bending-to-straightening behavior among local crystallization areas 2 (scale bar: 100 μm) (MP4)
