# Supplementary material for: Amorphous–Crystalline Solid Transformation-Induced Self-Actuation of Bending-to-Straightening Behavior via Helical Deformation
Source: Cryst Growth Des. 2025 Nov 5;25(22):9836–48. doi: 10.1021/acs.cgd.5c01103 (PMC12636014; doi:10.1021/acs.cgd.5c01103)
Supplement: Supplementary file 1 [file cg5c01103_si_001.pdf]

Supplementary Information

Amorphous-crystalline Solid Transformation Induced Self-actuation of Bending-to-straightening Behavior via Helical Deformation

Xintong Meng[1], Yifan Huang[2], Qun Song[1], Ruhuai Mei[1], Lin Tian[3], Corinna Willenberg[4], Fen Li[1], Cynthia Volkert[3], Philipp Vana[5], Qiyun Tang[2], Ping Shao[6], Xun Wang[7]* and Kai Zhang[1,8]**

[1] Sustainable Materials and Chemistry, Department of Wood Technology and Wood-based Composites, University of Göttingen, Büsgenweg 4, 37077 Göttingen, Germany.

[2] Key Laboratory of Quantum Materials and Devices of Ministry of Education, School of Physics, Southeast University, 211189 Nanjing, China

[3] Institute of Materials Physics, University of Göttingen, Friedrich-Hund-Platz. 1, 37077 Göttingen, Germany

[4] Institute of Inorganic Chemistry, University of Göttingen, Tammannstr. 4, 37077 Göttingen, Germany

[5] Institute of Physical Chemistry, University of Göttingen, Tammannstr. 6, 37077 Göttingen, Germany

[6] Department of Food Science and Technology, Zhejiang University of Technology, 310014 Hangzhou, China

[7] Faculty of Chemistry, Tsinghua University, 100084, Beijing, China

[8] Biotechnology Center (Biotechnikum), University of Göttingen, Büsgenweg 2, 37077 Göttingen, Germany

The PDF file includes:

Methods
Supplementary Schemes 1 to 2
Supplementary Figs. 1 to 47
Supplementary Tables 1 to 2
Supplementary Note 1
References

Other Supplementary Materials for this manuscript include the following:

Supplementary Movies 1 to 4

Methods

Preparation of 2,3,4,6-tetra-O-acetyl-β-D-glucopyranosyl azide (GluA)

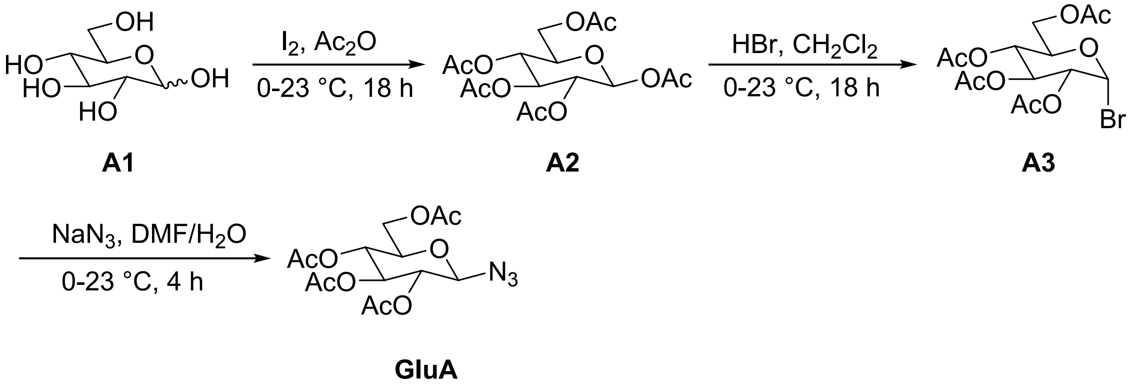

Scheme 1. Synthesis pathway of 2,3,4,6-tetra-O-acetyl-β-D-glucopyranosyl azide (GluA).

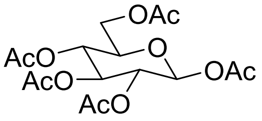

A2

(2S,3R,4S,5R,6R)-6-(acetoxymethyl) tetrahydro-2H-pyran-2,3,4,5-tetrayl tetraacetate (A2)

D-glucose (50 g, 250 mmol) was added slowly in several portions to Ac_2O (300 mL) at 0 °C, then I_2 (1.0 g, 1.5 mol %) was added slowly. The mixture was allowed to reach ambient temperature gradually and stirred overnight. Saturated $Na_2S_2O_3$ (100 mL) was added to the mixture and stirred for 5 min, during which time the solution changed from orange to colorless. CH_2Cl_2 (500 mL) and H_2O (200 mL) were added to the mixture; the organic phase was washed extensively with H_2O until almost neutral, dried with Na_2SO_4, and concentrated in vacuo. Colorless oil (125 g, quantitative) was obtained which was solidified to a white solid in the fridge. The crude product was directly used without purification for the next step.

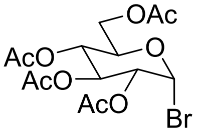

A3

(2R,3R,4S,5R,6R)-2-(acetoxymethyl)-6-bromotetrahydro-2H-pyran-3,4,5-triyl triacetate (A3)

Acetylated glucose 2 (166 mmol, crude product from the previous step) was dissolved in CH_2Cl_2 (200 mL) and HBr (200 mL, 33 wt.% in AcOH) was added slowly at 0 °C. The mixture was allowed to reach ambient temperature gradually and stirred for an additional 18 h. Then, H_2O (150 ml) and CH_2Cl_2 (100 mL) were added and the organic phase was washed with H_2O extensively until almost neutral. After further washed with saturated Na_2CO_3 and brine, the organic solution was dried over Na_2SO_4, filtrated, dried in vacuo, and purified by recrystallization from n-

hexane/EA to afford a white solid (83.7 g, 61% for two steps). ^{1}H NMR (400 MHz, $CDCl_3$) δ = 6.62–6.56 (m, 1H), 5.59–5.46 (m, 1H), 5.19–5.10 (m, 1H), 4.86–4.78 (m, 1H), 4.44–4.21 (m, 2H), 4.18–4.00 (m, 1H), 2.11 (s, 3H), 2.06 (s, 3H), 2.04 (s, 6H). ^{13}C NMR (100 MHz, $CDCl_3$) δ = 170.4 (C_q), 169.7 (C_q), 169.6 (C_q), 169.3 (C_q), 86.5 (CH), 72.0 (CH), 70.4 (CH), 70.0 (CH), 67.0 (CH), 60.8 (CH_2), 20.5 (CH_3), 20.5 (CH_3), 20.5 (CH_3), 20.4 (CH_3).

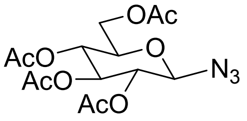

GluA

(2R,3R,4S,5R,6R)-2-(Acetoxymethyl)-6-azidotetrahydro-2H-pyran-3,4,5-triyl triacetate (GluA)

D-glucopyranosyl bromide A3 (49.18 g, 120 mmol) was dissolved in DMF (150 mL). Then H_2O (25 mL) and NaN_3 (11.4 g, 175.3 mmol, 1.5 eq) were added at 0 °C. The mixture was allowed to be gradually warmed to ambient temperature and stirred for 18 h. Workup: H_2O (150 mL) and EtOAc (300 mL) were added. The organic layer was washed with H_2O (2×100 mL) and brine (1×100 mL), dried with Na_2SO_4, and concentrated in vacuo. During the concentration, large amount of white precipitate formed which could be further purified by recrystallization in EtOAc/nHexane to afford the final product S4 as a white solid (32.2 g, 72%). Mp：126-127 °C. ^{1}H NMR (400 MHz, $CDCl_3$) δ = 5.29–5.17 (m, 1H), 5.11 (t, J = 9.7 Hz, 1H), 4.96 (tt, J = 9.4, 1.1 Hz, 1H), 4.66 (dd, J = 8.9, 1.8 Hz, 1H), 4.28 (dd, J = 12.6, 4.6 Hz, 1H), 4.17 (dt, J = 12.6, 1.7 Hz, 1H), 3.86–3.76 (m, 1H), 2.10 (s, 3H), 2.08 (s, 3H), 2.03 (s, 3H), 2.01 (s, 3H). ^{13}C NMR (100 MHz, $CDCl_3$) δ = 170.5 (C_q), 170.0 (C_q), 169.2 (C_q), 169.1 (C_q), 87.8 (CH), 73.9 (CH), 72.5 (CH), 70.6 (CH), 67.8(CH), 61.6 (CH_2), 20.6 (CH_3), 20.5 (CH_3), 20.5 (CH_3), 20.5 (CH_3). ESI-HRMS: m/z calcd. for $C_{14}H_{19}N_3O_9Na$ $[M+Na]^+$: 396.09, found 396.10.

Preparation of 2,3,4,6-tetra-O-acetyl-β-D-galactopyranosyl azide (GalA)

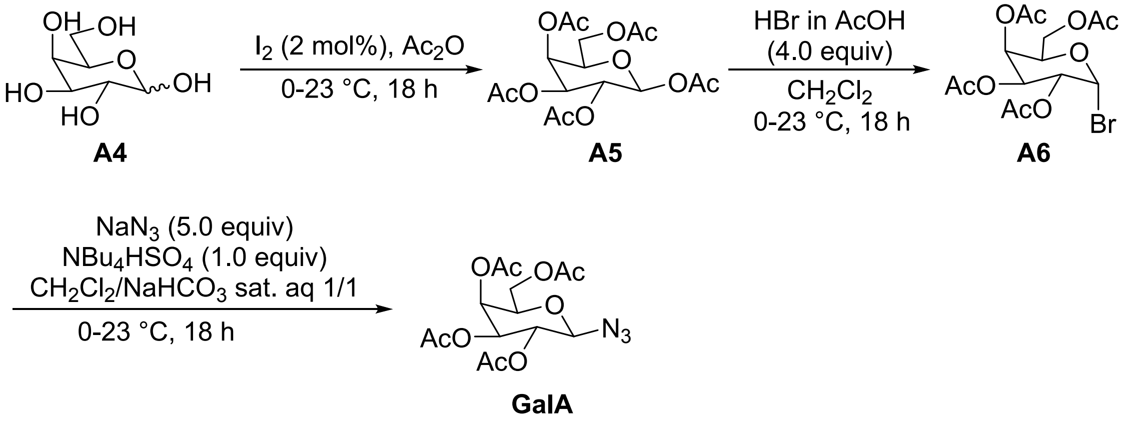

Scheme 2. Synthesis pathway of 2,3,4,6-tetra-O-acetyl-β-D-galactopyranosyl azide (GalA).

The procedure of A5 and A6 was followed as same as A2 and A3.

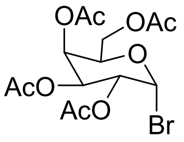

A6

(2R,3S,4S,5R,6R)-2-(Acetoxymethyl)-6-bromotetrahydro-2H-pyran-3,4,5-triyl triacetate (A6)

The same procedure was followed as D-glucopyranosyl bromide. Pale yellow oil. ^{1}H NMR (400 MHz, $CDCl_3$) δ = 6.69 (d, J = 4.0 Hz, 1H), 5.51 (d, J = 3.3 Hz, 1H), 5.39 (dd, J = 10.6, 3.4 Hz, 1H), 5.04 (dd, J = 10.7, 3.8 Hz, 1H), 4.48 (t, J = 6.6 Hz, 1H), 4.18 (dd, J = 11.4, 6.3 Hz, 1H), 4.10 (dd, J = 11.0, 7.0 Hz, 1H), 2.14 (s, 3H), 2.10 (s, 3H), 2.05 (s, 3H), 2.00 (s, 3H). ^{13}C NMR (100 MHz, $CDCl_3$) δ = 170.2 (C_q), 170.0 (C_q), 169.8 (C_q), 169.7 (C_q), 88.1 (CH), 71.0 (CH), 67.9 (CH), 67.7 (CH), 66.9 (CH), 60.7 (CH_2), 20.6 (CH_3), 20.5 (CH_3), 20.5 (CH_3), 20.4 (CH_3).

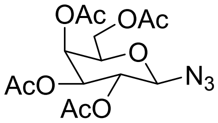

GalA

(2R,3S,4S,5R,6R)-2-(Acetoxymethyl)-6-azidotetrahydro-2H-pyran-3,4,5-triyl triacetate (S2)

D-galacopyranosyl bromide A6 (4.57 g, 11.1 mmol) was dissolved in CH_2Cl_2 (25 mL). Then, saturated aqueous $NaHCO_3$ (25 mL) NBu_4HSO_4 (3.7 g, 11.11 mmol, 1.0 eq) and NaN_3 (3.6 g, 55.6 mmol, 5.0 eq) were added at 0 °C. The mixture was allowed to be gradually warmed to ambient temperature and stirred for 18 h. Workup: H_2O (150 mL) and EtOAc (300 mL) were added. The organic layer was washed with H_2O (2×100 mL) and brine (1×100 mL), dried with Na_2SO_4, and concentrated in vacuo. During the concentration, large amount of white precipitate formed which could be further purified by recrystallization in EtOAc/nHexane to afford the final product S2 as a white solid (3.49 g, 84%). Mp：96-97 °C. ^{1}H NMR (400 MHz, $CDCl_3$) δ = 5.44 (dd, J = 3.3, 1.4 Hz, 1H), 5.18 (dd, J = 10.3, 8.8 Hz, 1H), 5.08– 5.02 (m, 1H), 4.61 (d, J = 8.7 Hz, 1H), 4.25–4.11 (m, 2H), 4.03 (t, J = 6.5 Hz, 1H), 2.18 (s, 3H), 2.10 (s, 3H), 2.07 (s, 3H), 2.00 (s, 3H). ^{13}C NMR (100 MHz, $CDCl_3$) δ = 170.3 (C_q), 170.1 (C_q), 170.0 (C_q), 169.3 (C_q), 88.3 (CH), 72.8 (CH), 70.7 (CH), 68.0 (CH), 66.8 (CH), 61.2 (CH_2), 20.6 (CH_3), 20.6 (CH_3), 20.6 (CH_3), 20.5 (CH_3). ESI-HRMS: m/z calcd. for $C_{14}H_{19}N_3O_9Na$ $[M+Na]^+$: 396.09, found 396.10.

SC-XRD measurement

Single crystals of the investigated compounds were coated with a trace of Fomblin Y oil (LOT: MKCK7738) and evaluated using a Nikon SMZ 1500 stereomicroscope under polarized light. After selecting a crystal, it was mounted on a MiTeGen micromount with sizes of 20, 50, or 200 µm and placed on a DB three-circle goniometer. The crystal was surrounded by a stream of nitrogen gas to ensure an oxygen- and moisture-free environment and to maintain a constant temperature. The measurements were conducted at 100(2) K. The X-ray beam was generated using

an Incoatec IμS microfocus source (Mo Kα, λ=0.71073 Å) equipped with an Incoatec Quazar mirror optic. The diffracted reflection intensities were recorded using either a SMART APEXII CCD detector or a Montel Photon 3C7 detector. APEX4 (Version v2021.4-0 (8)) was used for operating the diffractometer, calculating a measurement strategy, and conducting an initial evaluation and processing of the data. The integration of reflection images was carried out using SAINT (Version V8.408[1]), which is integrated as a plugin in APEX. During further data processing, an absorption correction was applied using SADABS (Version 2016/2[2]). In XPREP (Version 2014/2[3]), the unit cells were then transformed to the highest symmetry, and a space group was determined based on E-value statistics and systematic absences. The structure was solved using SHELXT (Version 2018/2[4]) and refined with SHELXT (Version 2018/3) through the ShelXle GUI[5].

Crystal Explorer 21 was used to compute the electrostatic potential map and the pairwise interaction energy between the molecules within the crystal using the wave function calculated at the B3LYP/6-31G(d,p) level of theory[6]. The four energy components, electrostatic (E ele), polarization (E pol), dispersion (E dis) and exchange–repulsion (E rep), were obtained using the wave function calculated at the B3LYP/6-31G(d,p) level of theory.

Cryo-EM measurement

Cryo-EM were carried out with applied 3-μl sample was to freshly glow-discharged Quantifoil Cu R1.2/1.3, 200 mesh grids, which were plunge-frozen with a Vitrobot Mark IV (Thermo Fisher Scientific) at 4 °C with 100% humidity. The grids were loaded to a Titan Krios G4 (Thermo Fisher Scientific) operating at 300 kV with parallel beam illumination for TEM (transmission electron microscope) imaging. Imaging mode was first used to locate the position of the crystals. Diffraction patterns of the crystals were acquired on a CMOS based Ceta-M detector (Thermo Fisher Scientific).

Raman spectra analysis

The polarized Raman spectra were recorded via LabRAM HR Evolution (Horiba France SAS) system. A 532 nm laser was applied. The acquisition time was 20 seconds, and each individual spectrum was the accumulation of 4 scans. Ensure the special resolution, hole size was kept at 100 μm. ½ λ waveplate was adopted to facilitate the angular dependence with steps of 10°.

CD spectra analysis

Circular Dichroism (CD) spectra were obtained using a JASCO J-1700 CD Spectrometer (JASCO Corporation, Japan) with a wavelength range of 200 to 400 nm. Signals for CD, LD, HTV, and Abs were collected. The beam size is around 2 mm. CD analysis was performed individually on the GluA solution and aligned straight crystalline rectangular hollow tubes, with three replicates for each sample. Interval CD spectroscopic measurements were performed in-situ, spanning a wavelength range of 200 to 350 nm, to observe the complete self-assembly process of GluA from solution to a suspension of straight crystalline rectangular hollow tubes. These measurements comprised two distinct types: temperature-interval and time-interval. For the temperature interval measurements, seven points were selected ranging from 353.15 K to 277.15 K, with a waiting time of 60 s between each interval. Time interval measurement was performed at 277.15 K with 5 s as interval.

AFM measurement

AFM characterization was performed in the dry state using a Multimode 8 AFM (Bruker, Karlsruhe, Germany) with a NanoScope V controller in an ambient environment. Soft probes (ScanAsyst-Air-HR, Bruker) with a nominal spring constant of 0.4 $N \cdot m^{-1}$, a nominal resonance frequency of 70 kHz and a nominal tip radius of 2 nm were used. The ScanAsyst-HR in Air mode was used as the imaging mode, with a scan rate of 0.97 Hz and a resolution of 512 × 512 samples per line.

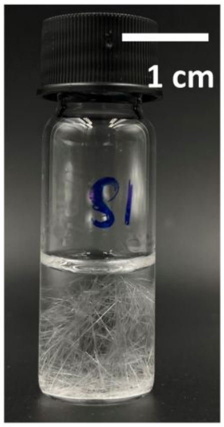

Supplementary Figure 1. Straight crystalline rectangular hollow tubes in MeOH /water solution after self-assembly.

a

b

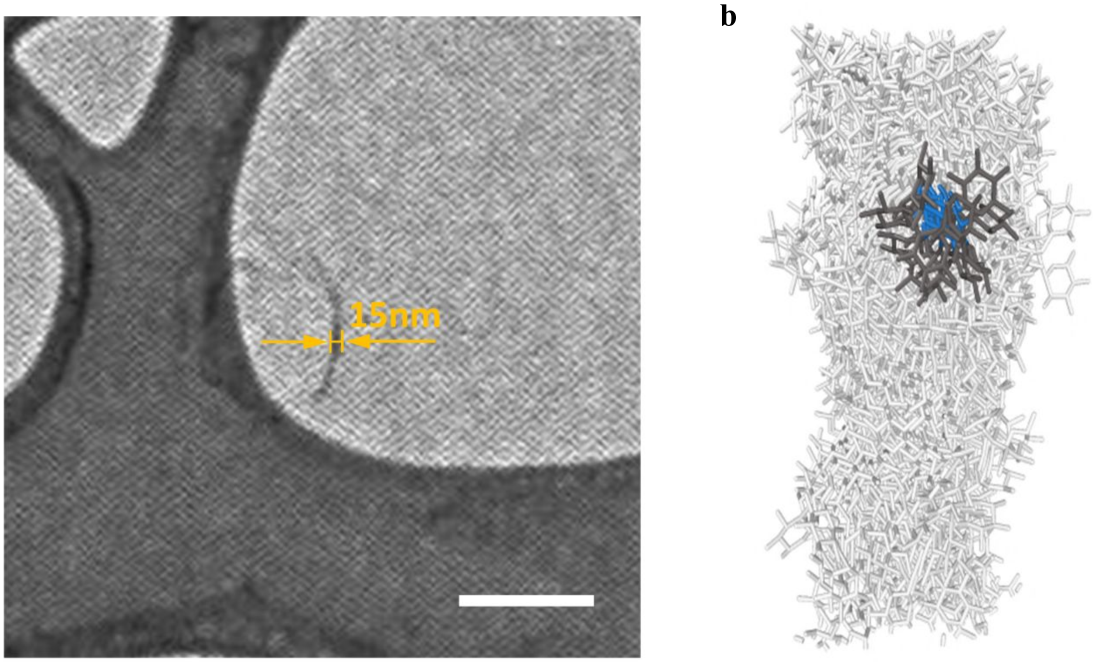

Supplementary Figure 2. (a) TEM image of the molecular aggregation. (b) MD simulation of the molecular aggregation. Scale bar: 200 nm.

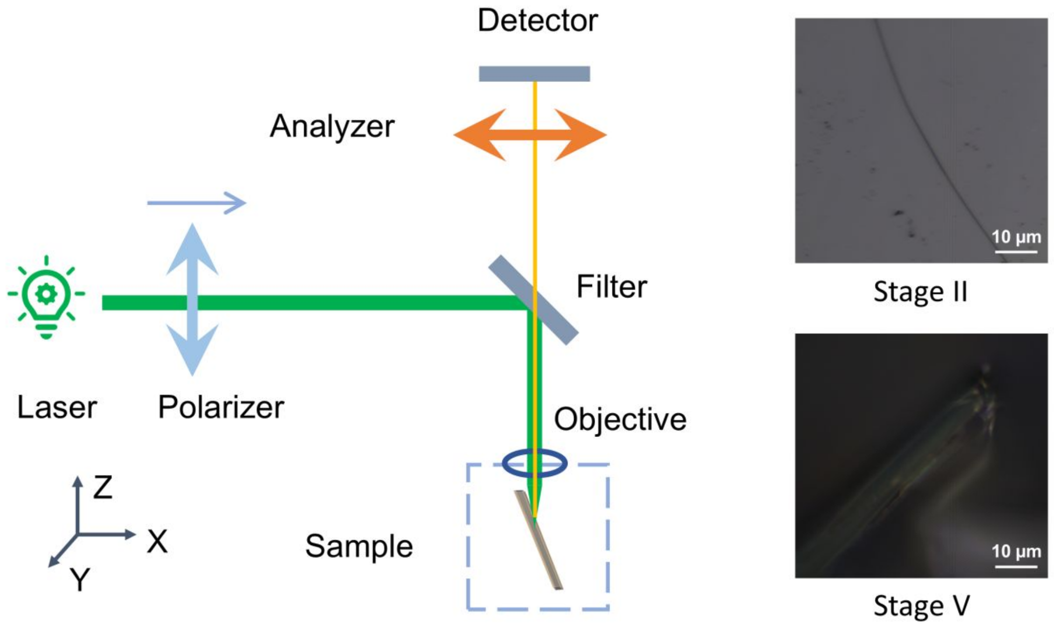

Supplementary Figure 3. Schematic of the polarized Raman spectroscopy setup of the crystallographic characteristics of Stage II and Stage V.

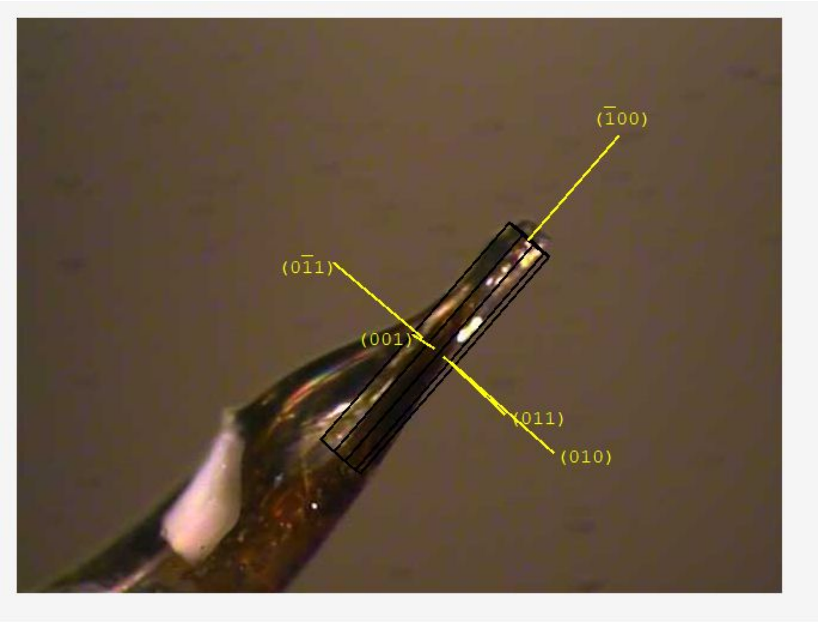

Supplementary Figure 4. A bulk orthorhombic crystal of GluA face-indexed with SC-XRD indicating the faces of (011), (001), (010), and their parallel counterparts.

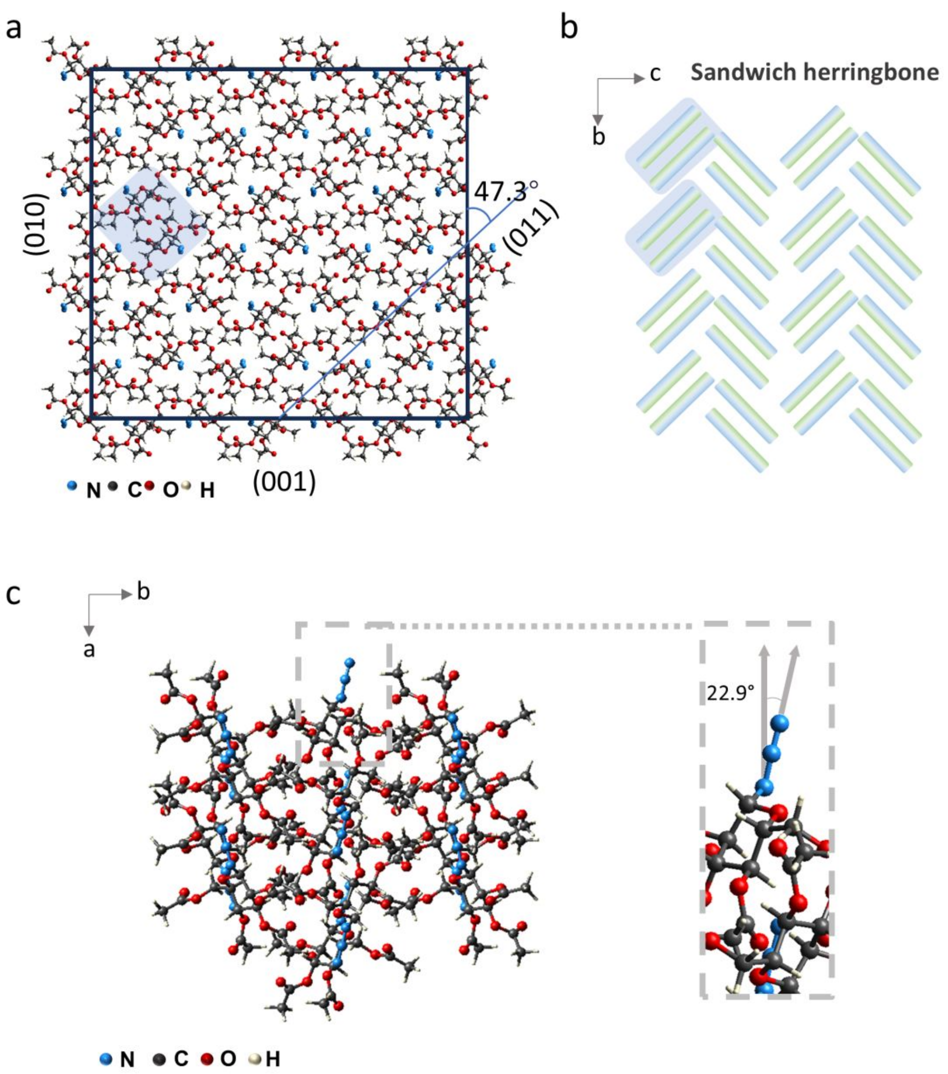

Supplementary Figure 5. (a) bc view of the extended molecular packing. (b) illustration of sandwich herringbone stacking. (c) ab view of the extended molecular packing.

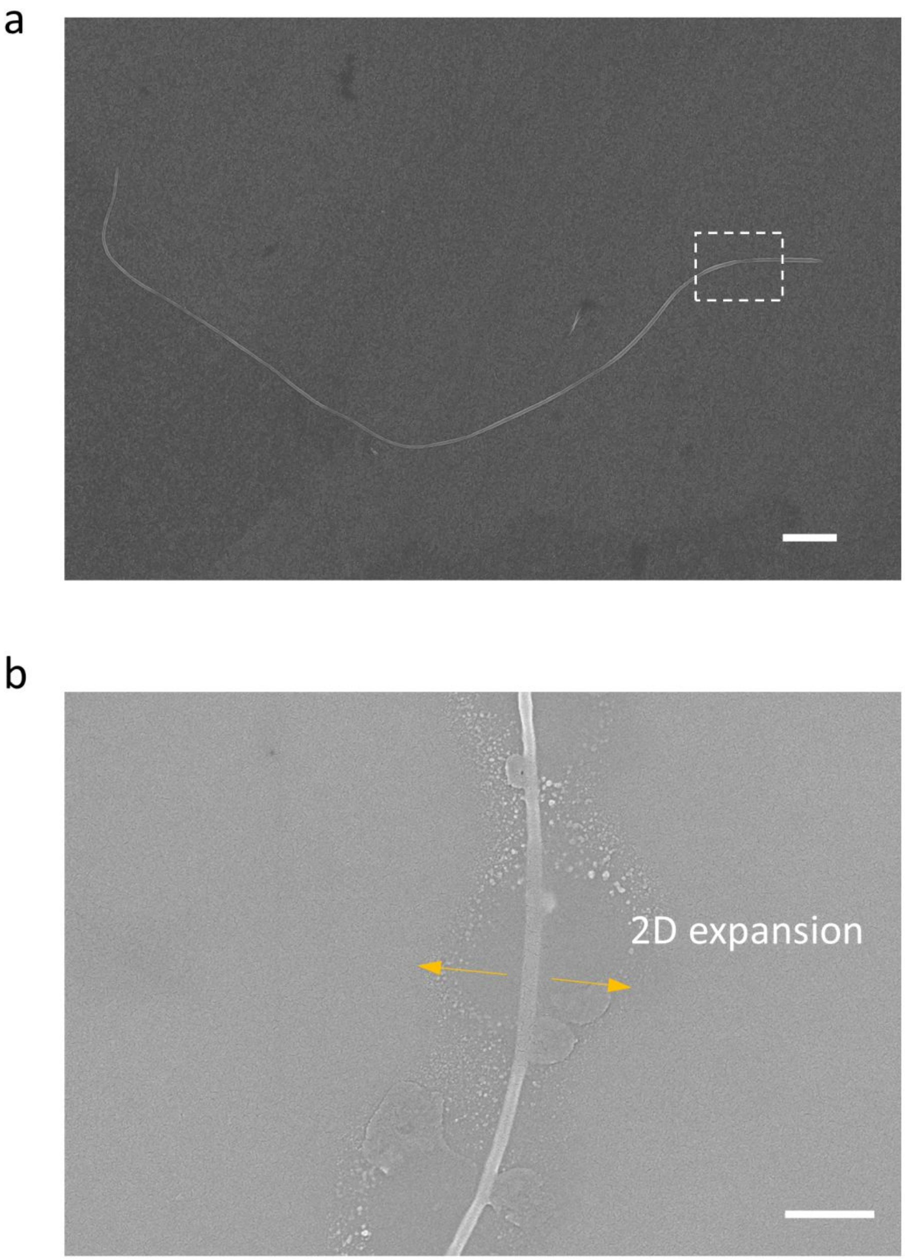

Supplementary Figure 6. (a) SEM image of the bending intermediate between Stage I and Stage II characterized by the uneven distributed diameter. (b) SEM image illustration of the 2D expansion in Stage II. Scale bar: 2 μm, 600 nm.

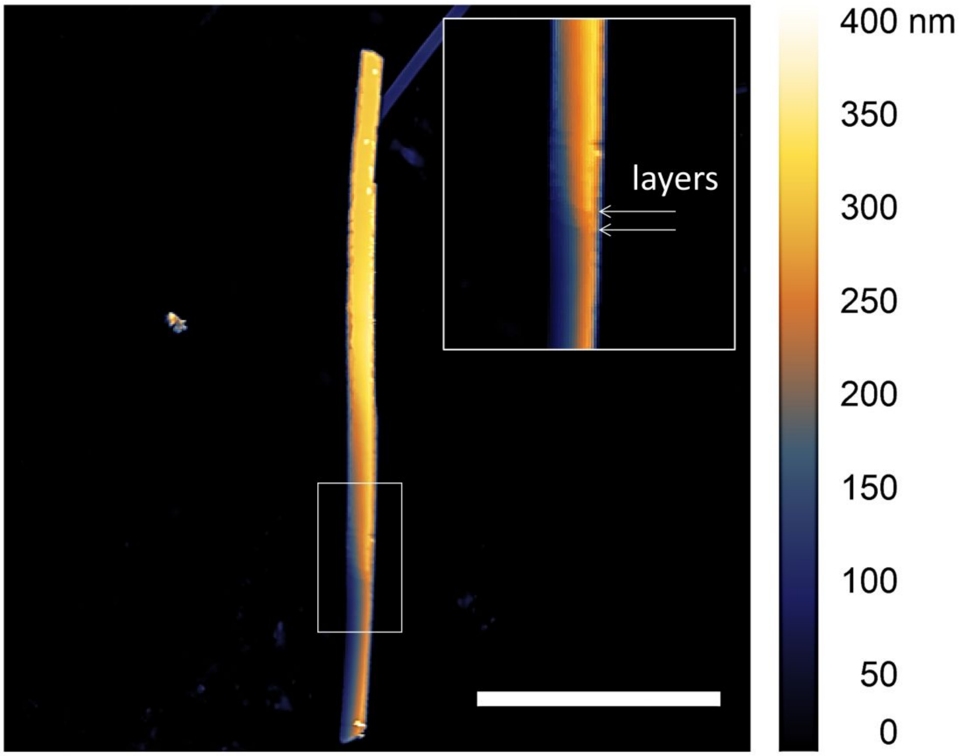

Supplementary Figure 7. AFM image of the helical deformation between Stage II and Stage III. Scale bar: 5 μm.

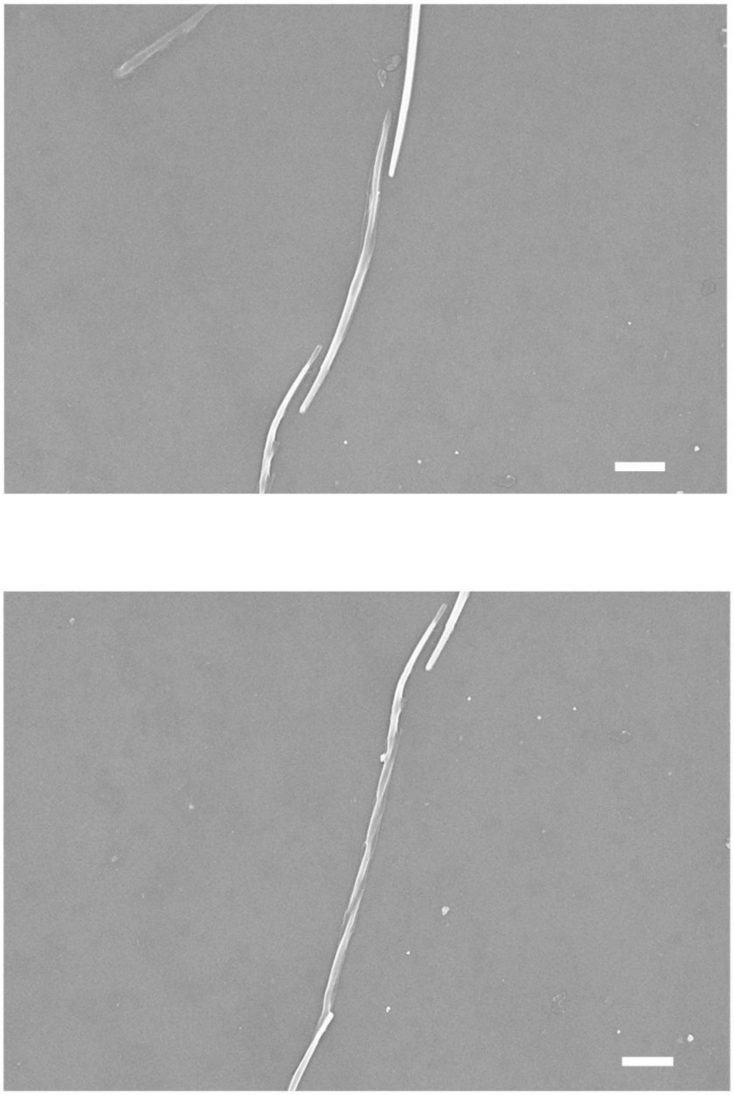

Supplementary Figure 8. SEM image of the helical deformation of Stage III. Scale bars: 2 μm.

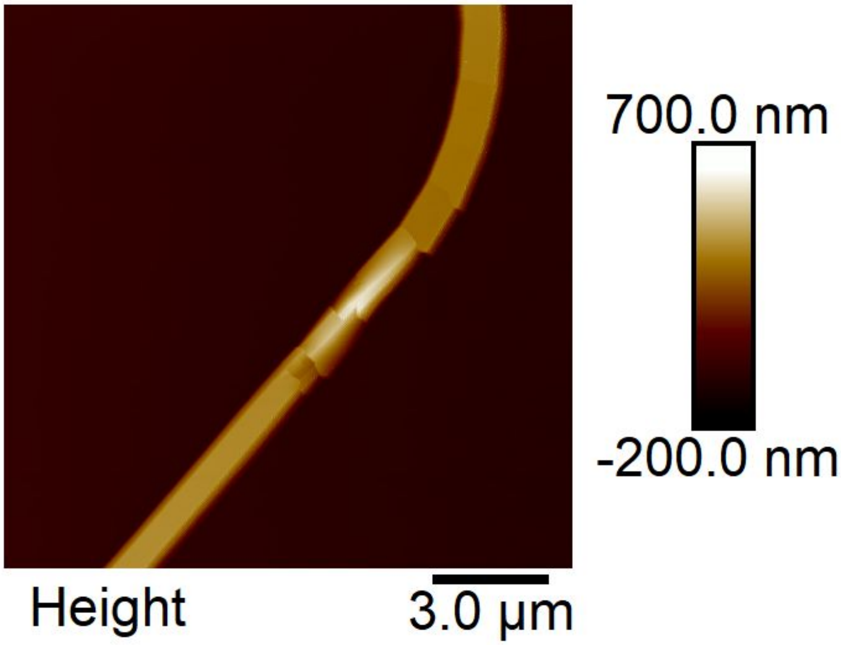

Supplementary Figure 9. AFM image of the crack-twist structure at Stage IV.

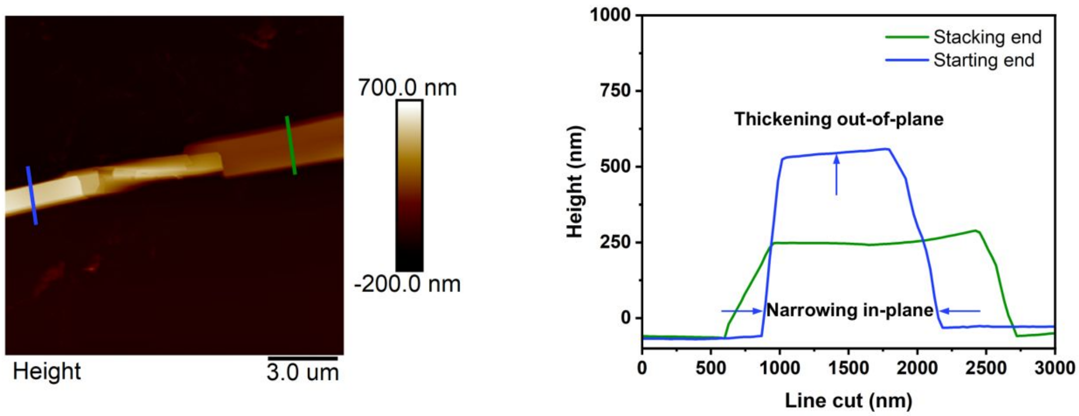

Supplementary Figure 10. AFM image of Stage IV in Figure 1i and cut profiles of the corresponding areas. Arrows illustrate the thickening in the out-of-plane direction and narrowing in the in-plane direction.

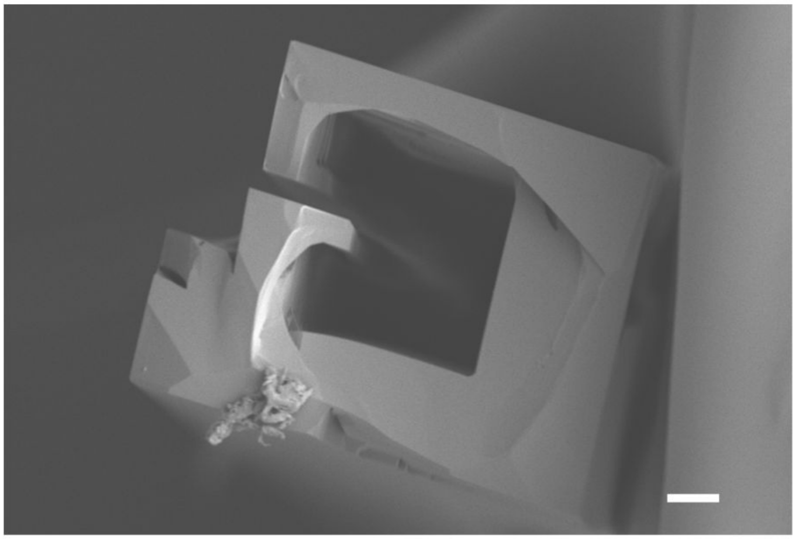

Supplementary Figure 11. SEM image of the cross-section of straight crystalline rectangular hollow tube in Stage V. Scale bar: 20 μm.

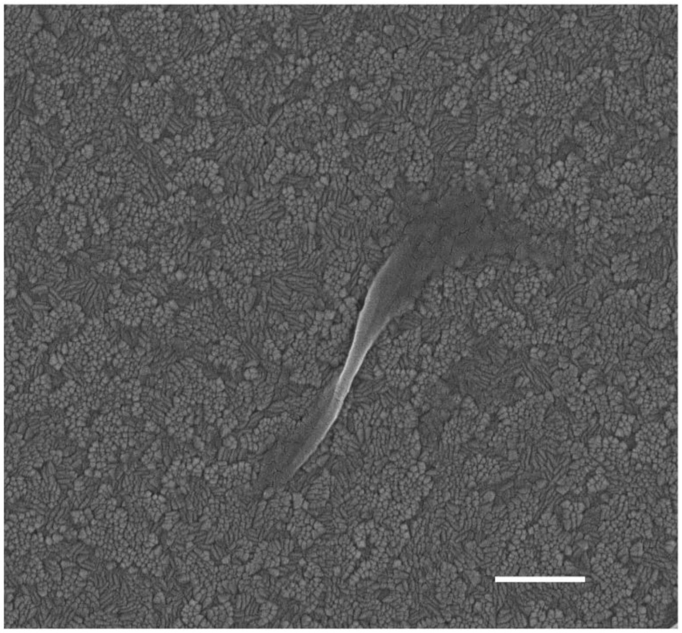

Supplementary Figure 12. Intermediate of independent twist captured by SEM. ITO slide was adopted as substrate. Scale bar: 400 nm.

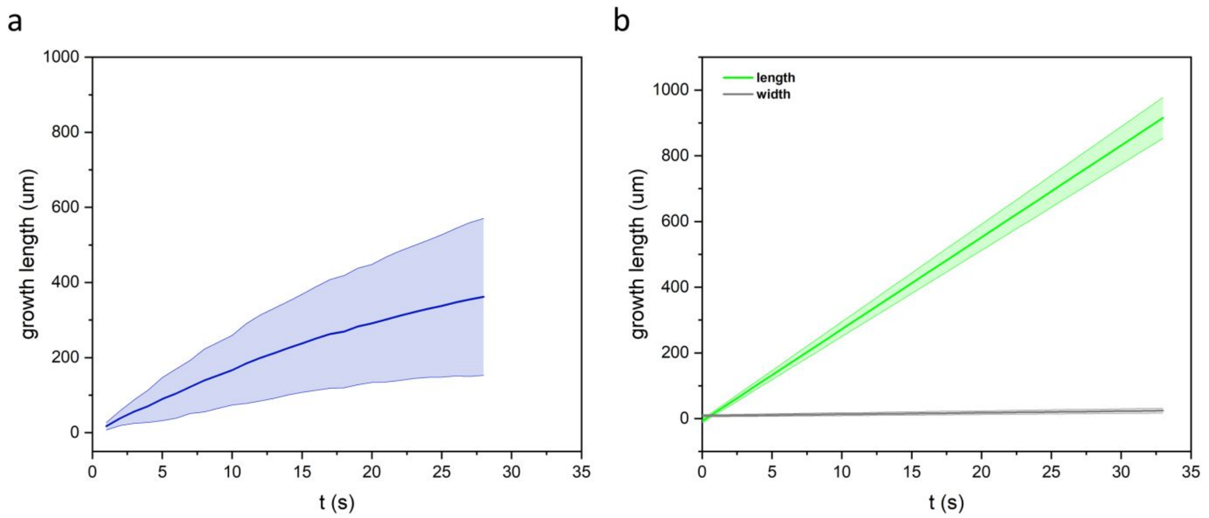

Supplementary Figure 13. (a) Growth rate at longitudinal direction of bending to straightening motion from Stage III to Stage IV. (b) Growth rate at longitudinal direction length and radius expansion width of Stage V. The growth length was obtained by quantitative analysis of the crystal tip position using software (tracker) for video analysis.

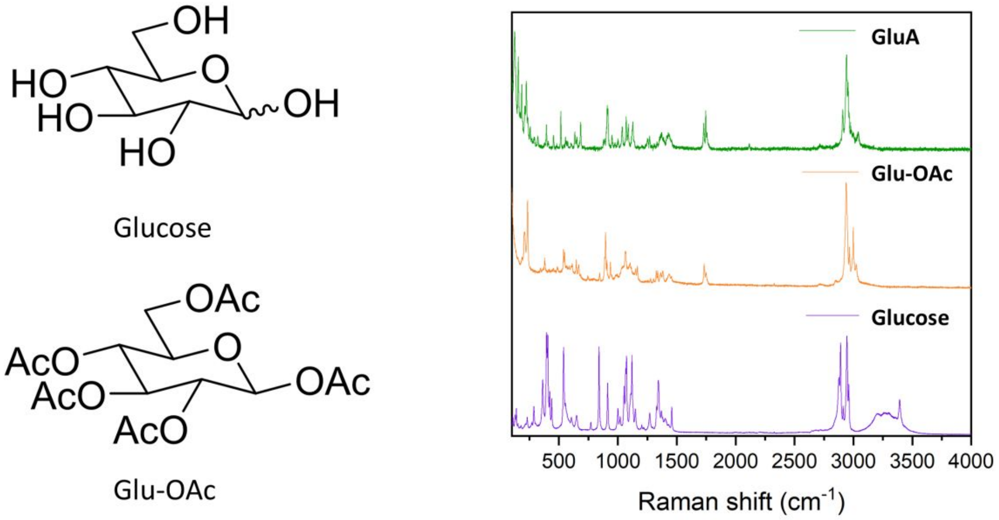

Supplementary Figure 14. Chemical structure of glucose and penta-O-acetyl-β-D-glucopyranose (Glu-OAc). Raman spectra of GluA, Glu-OAc, and glucose.

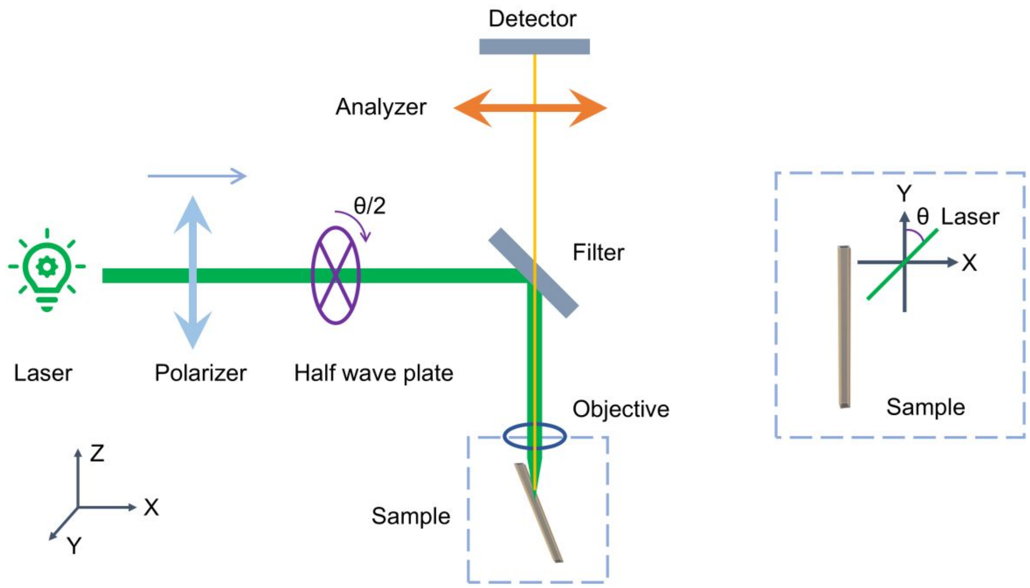

Supplementary Figure 15. Schematic of the polarized Raman spectroscopy setup for the angular-dependent Raman spectra.

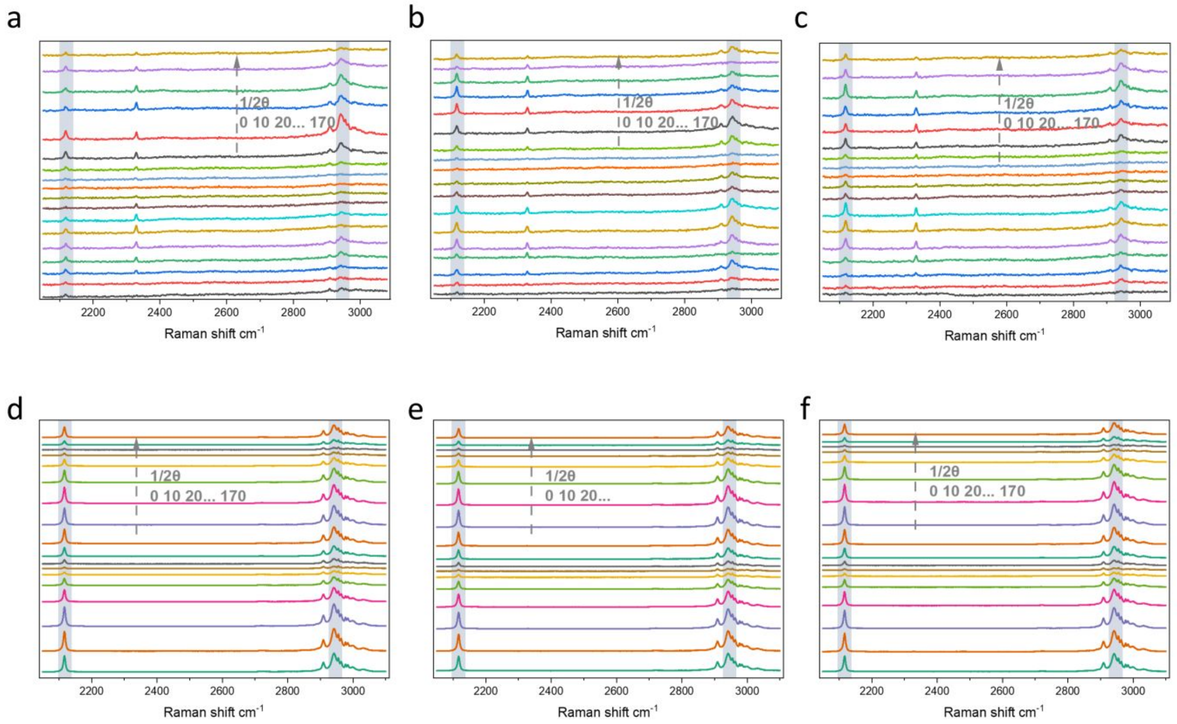

Supplementary Figure 16. Angle-resolved Raman spectra of the three-spot selected from the twist in Stage III: (a) i after twist, (b) ii on the twist, and (c) iii before twist. The plots are arranged from bottom to top, spanning 1/2 θ from 0° to 170° with a 10° step increment. Angle-resolved Raman spectra of the three-spot selected from the straight crystalline rectangular hollow tube in Stage V: (d) i section, (e) ii section, and (f) iii section. The plots are arranged from bottom to top, spanning 1/2 θ from 0° to 170° with a 10° step increment.

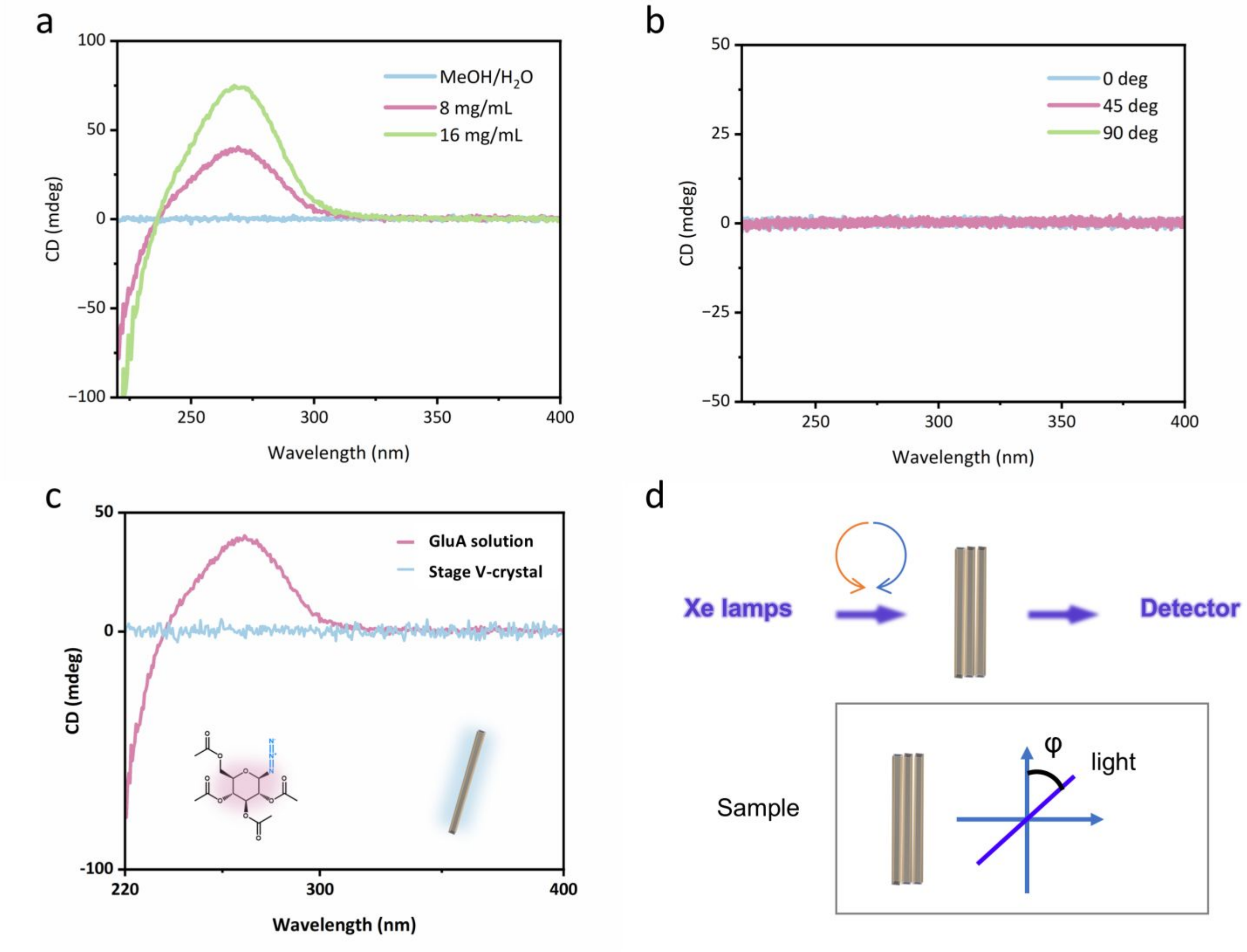

Supplementary Figure 17. (a) CD spectrum of GluA solution in MeOH/H_2O with different concentrations. (b) CD spectrum of the straight crystalline rectangular hollow tube with different azimuth angle (φ). (c) CD spectrum of GluA solution and the straight crystalline rectangular hollow tube of Stage V. (d) CD set up of measuring the straight crystalline rectangular hollow tube. The tubes are arranged parallel to each other to enhance the detected intensity.

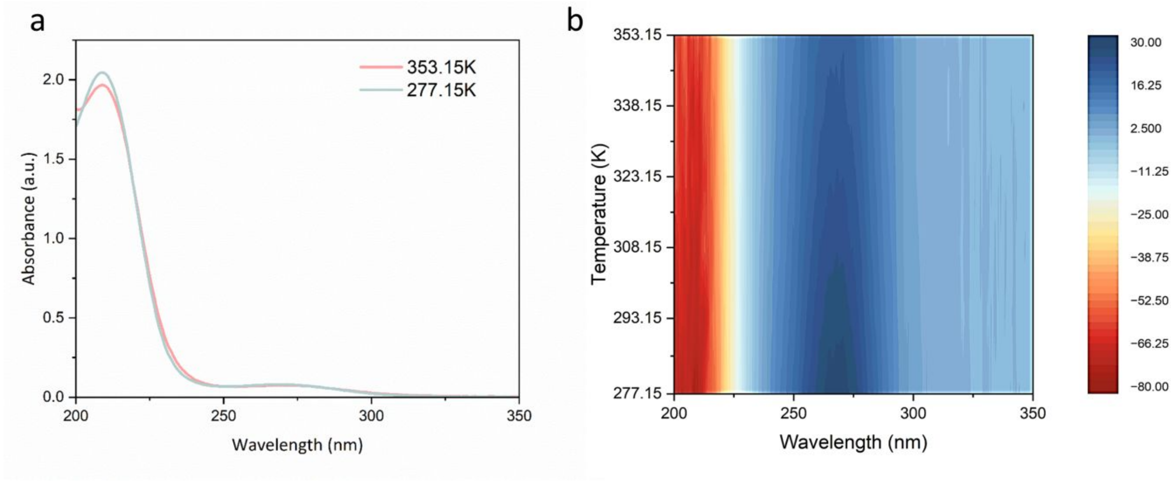

Supplementary Figure 18. (a) Absorbance spectrum of GluA solution under 353.15 K and 277.15 K. (b) Temperature interval CD spectrum of GluA in MeOH/H_2O from 353.15 K to 277.15 K.

a

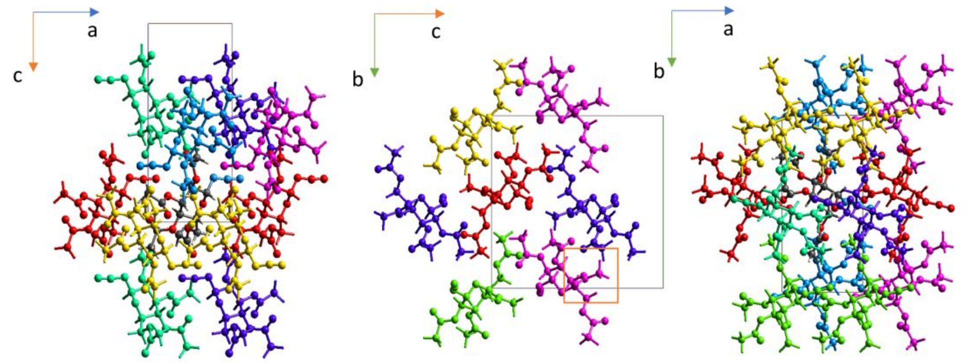

b

	N	Symop	R	Electron Density	E_ele	E_pol	E_dis	E_rep	E_tot
	2	x, y, z	7.30	B3LYP/6-31G(d,p)	-10.9	-5.3	-40.8	26.0	-35.0
	2	x+1/2, -y+1/2, -z	7.36	B3LYP/6-31G(d,p)	-20.9	-5.7	-41.8	33.2	-42.2
	2	x+1/2, -y+1/2, -z	10.01	B3LYP/6-31G(d,p)	-3.5	-1.9	-22.9	14.8	-16.0
	2	-x+1/2, -y, z+1/2	8.59	B3LYP/6-31G(d,p)	-13.5	-5.2	-27.8	22.5	-28.4
	2	-x, y+1/2, -z+1/2	9.10	B3LYP/6-31G(d,p)	-8.8	-2.0	-21.5	12.9	-21.6
	2	-x+1/2, -y, z+1/2	9.22	B3LYP/6-31G(d,p)	-13.0	-3.1	-23.3	14.2	-27.6
	2	-x, y+1/2, -z+1/2	12.14	B3LYP/6-31G(d,p)	-1.8	-0.3	-3.3	0.0	-5.0

Supplementary Figure 19. (a) Intermolecular energy calculation based on the molecular packing with the radius of 3.8 Å. (b) Table of the intermolecular energy. Calculations are executed by using the following equation:

$$E_{tot}=k_{ele}E_{ele}+k_{pol}E_{pol}+k_{dis}E_{dis}+k_{rep}E_{rep}$$

Where k_{ele}= 1.019, k_{pol}= 0.651, k_{dis}= 0.901, and k_{rep} =0.811.

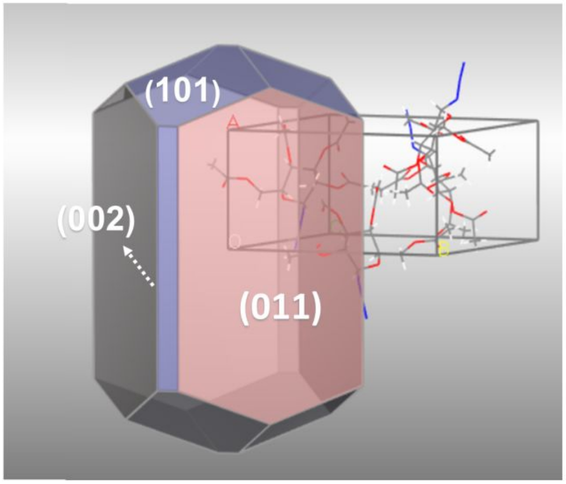

Supplementary Figure 20. Crystal morphology of GluA from prediction by BFDH theory.

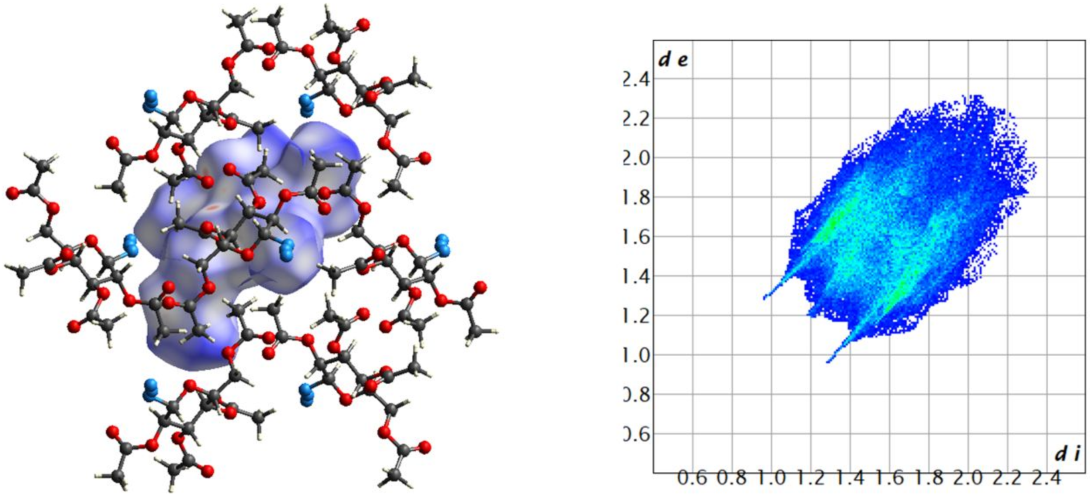

Supplementary Figure 21. Hirshfeld surfaces of GluA with d_{norm} plotted from -0.2501 (red) to 1.2911 (blue) Å, and 2D fingerprint plot. Drawings plotted using CrystalExplorer. The d_{norm}, which combines the internal d_i and external d_e distances from the surface to the nearest nucleus.

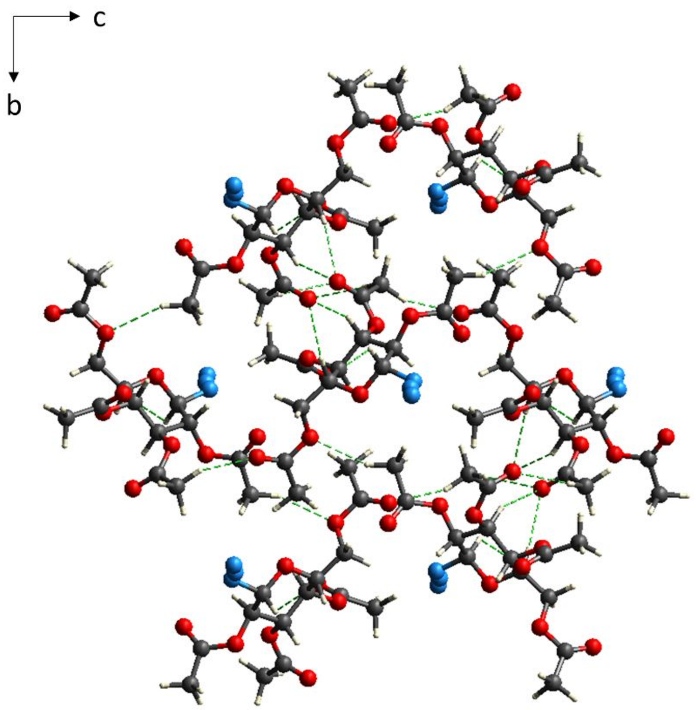

Supplementary Figure 22. Intermolecular hydrogen bonding network within molecular packing with radius of 3.8 Å.

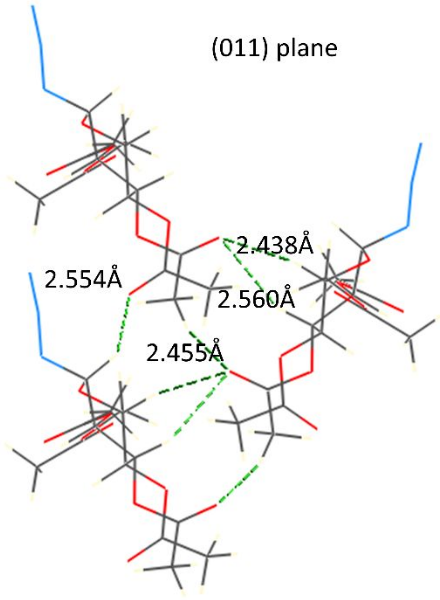

Supplementary Figure 23. Intermolecular hydrogen bonding within the building block dimers in the sandwich herringbone molecular packing. Hydrogen bonds: H (C5)···O (C3 C=O) 2.438 Å; H (C3)···O (C3 C=O) 2.560 Å; O (C3 C=O)···H (C3 CH_3) 2.455 Å.

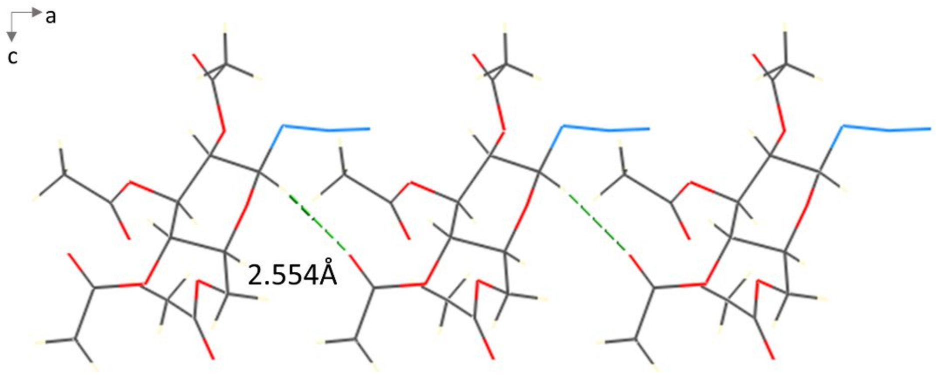

Supplementary Figure 24. Intermolecular hydrogen bonding packing unit along a axis. Hydrogen bond: H (C1)···O (C4 C=O) 2.544 Å.

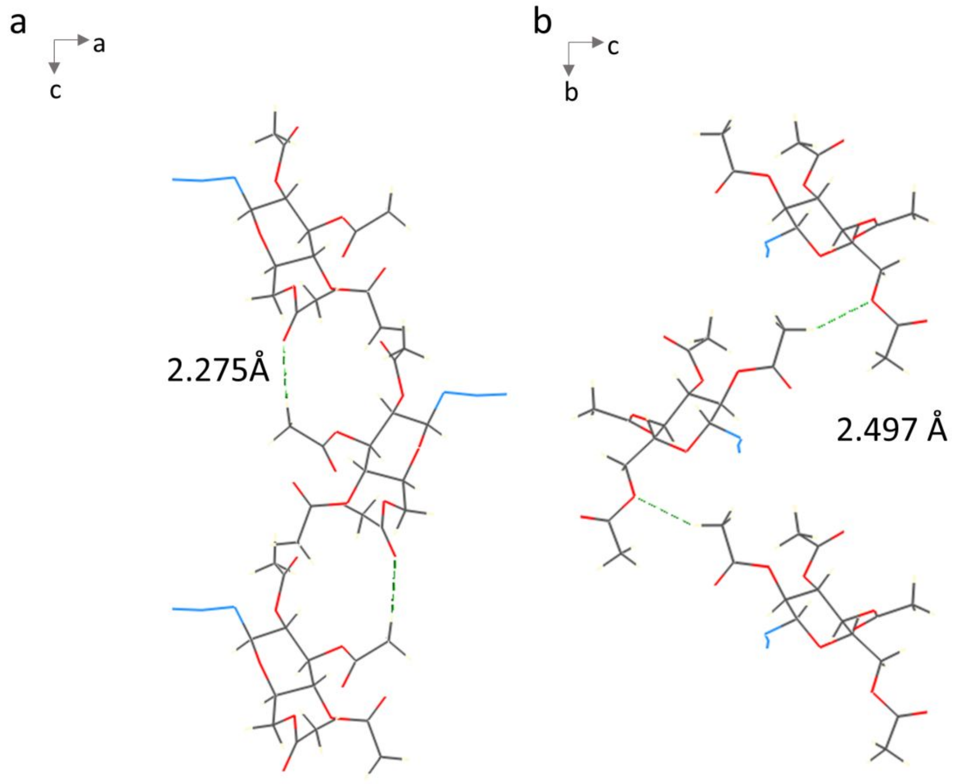

Supplementary Figure 25. (a) Intermolecular hydrogen bonding packing unit along c axis. Hydrogen bond: O (C6 C=O) ···H (C3 CH_3) 2.275 Å. (b) Intermolecular hydrogen bonding packing unit along b axis. Hydrogen bond: O (C6 C=O) ···H (C2 CH_3) 2.497 Å.

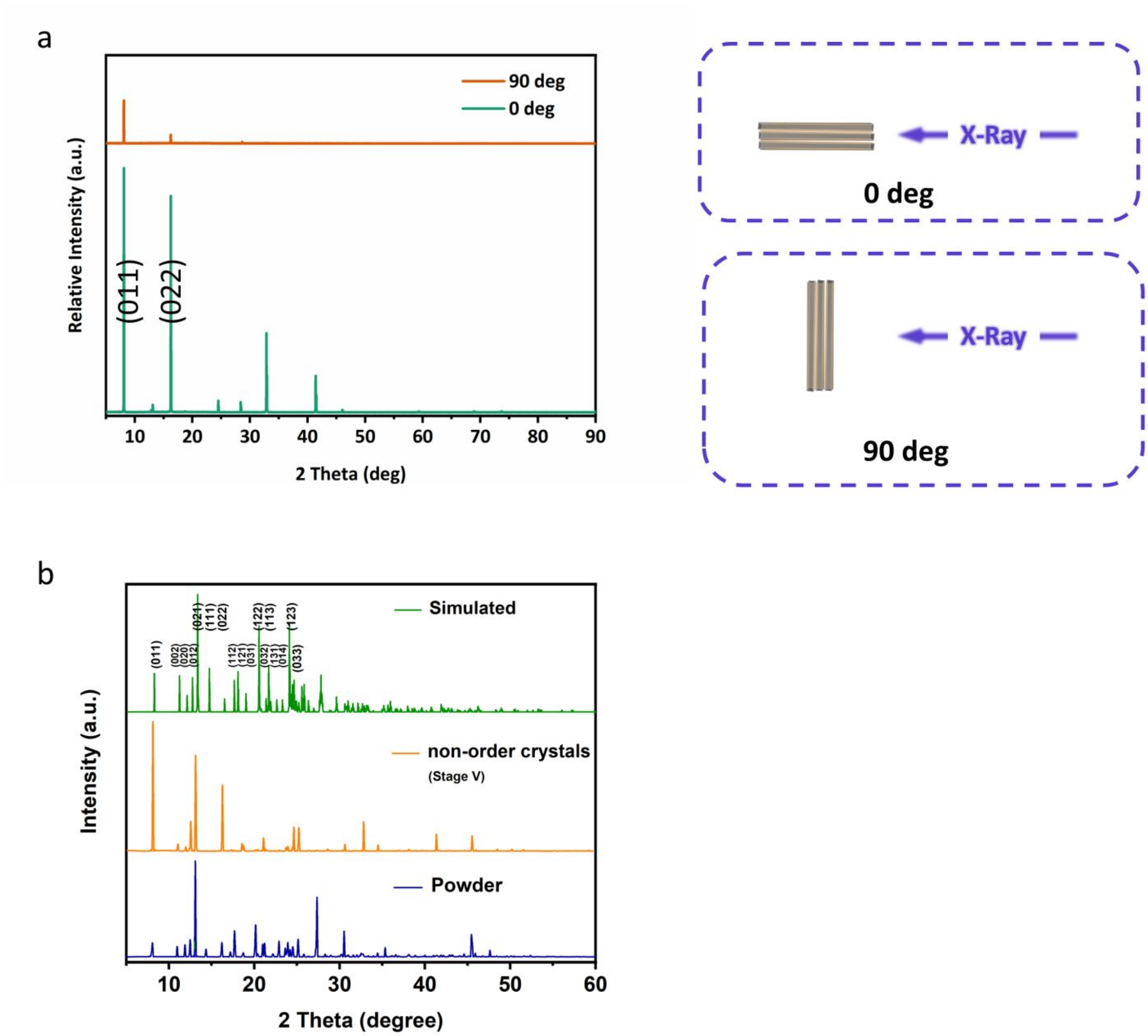

Supplementary Figure 26. (a) PXRD of aligned straight crystalline rectangular hollow tubes at Stage V with azimuth angles of 0° and 90° respect to the X-ray beam. (b) PXRD of simulated crystalline GluA, non-ordered crystals in Stage V, and GluA powder sample before self-assembly. Non-ordered sample consists of straight crystalline rectangular hollow tubes at Stage V, randomly oriented with respect to each other. Powder sample represents the freshly synthesized GluA after drying as the white solids.

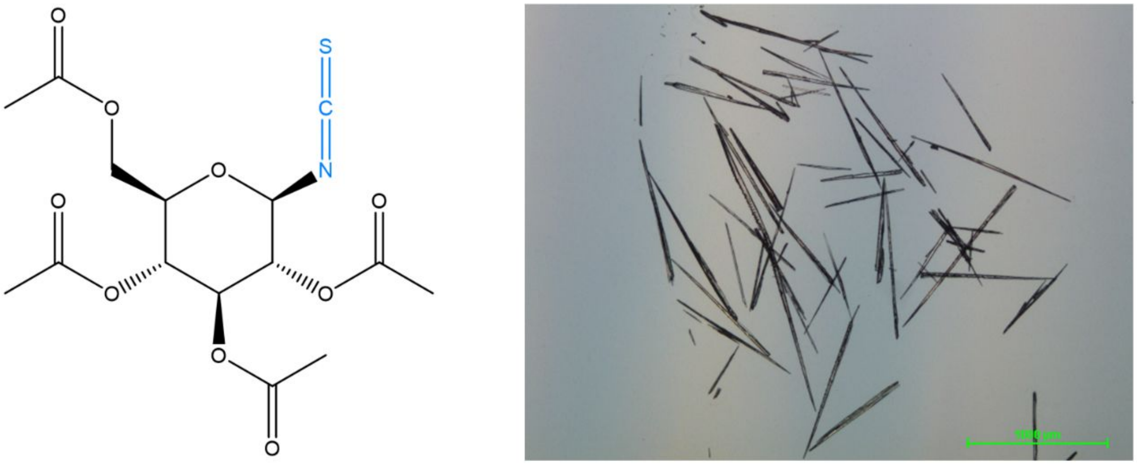

Supplementary Figure 27. Chemical structure of 2,3,4,6-tetra-O-acetyl-beta-D-glucopyranosyl-isothiocyanate (GluI) and the optical microscope image of resulting crystals. The self-assembled solids of GluI were obtained in the same way as for the preparation of crystals based on GluA.

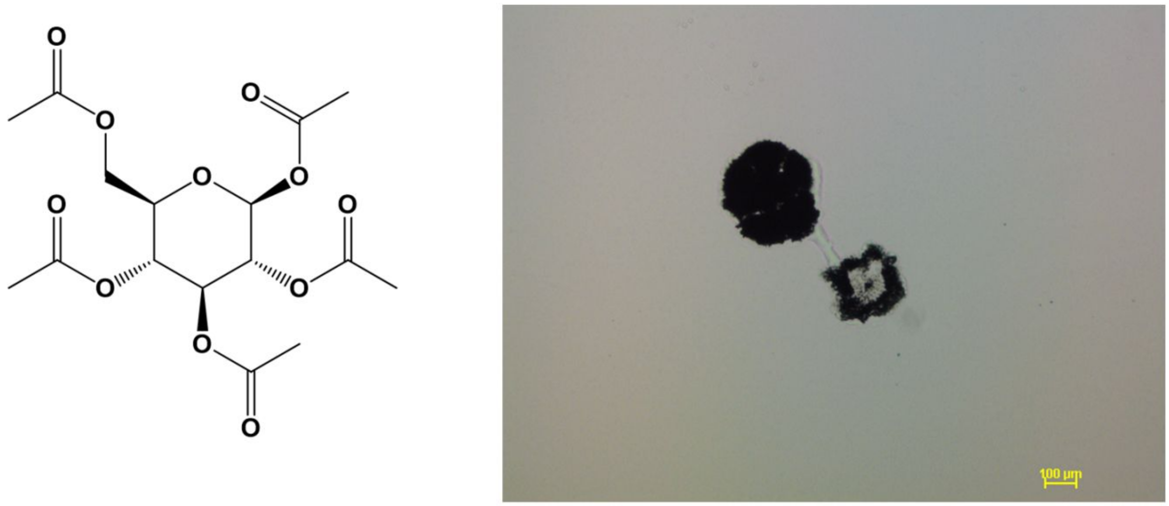

Supplementary Figure 28. Chemical structure of Glu-OAc and the optical microscope image of resulting self-assembled solid. The self-assembled solids of Glu-OAc were obtained through solvent evaporation from a solution (8 mg/mL in a 1:1 MeOH/H_2O mixture).

Supplementary Table 1. Crystallographic parameters of the single crystal GluA

**Name**	**GluA**
CCDC No.	2558837
formula	$C_{14}H_{19}N_3O_9$
FW	373.32
Crystal system	Orthorhombic
Space group	P2(1)2(1)2(1) #19
a [Å]	7.304
b [Å]	14.738
c [Å]	15.875
α [°]	90
β [°]	90
γ [°]	90
Volume / $Å^3$	1708.885805

Supplementary Table 2. Hydrogen bonds information

N	Position	Distance (Å)
1	H (C5) … O (C3_C=O)	2.461
2	H (C3) … O (C3_C=O)	2.567
3	H (C3_CH_3) … O (C3_C=O)	2.487
4	H (C1) … O (C4_C=O)	2.583
5	H (C3_CH_3) … O (C6_C=O)	2.295
6	H (C2_CH_3) ... O (C6_O-C-O)	2.493

Supplementary Note 1 – Coarse-Grained model and Molecular Dynamic simulations

A. Coarse-grained model of experimental system

To investigate the formation kinetics of GluA molecules at early stage, we employ a coarse-grained model of the experimental molecules to perform Molecular Dynamic (MD) simulations. The coarse-grained model of GluA molecule is compared to the real molecules as shown in Figure 34, which comprises two different types of beads: the central coarse-grained ring segments (R bead, the grey spheres in Figure 34b), which adsorb the O and H atoms onto the C atoms on sugar ring. The nitrogen-atoms connected to the ring are modeled as 3 beads with attractive interactions (N bead, see the blue spheres in Figure 34b). The other 4 hydrocarbon groups connected to the ring are modeled as R beads correspondingly. In our coarse-grained model, both N and R beads were assumed to have the same size of around 0.1 nm. To prevent deformation of the $-N_3$ group and the ring segment during the simulation, we treat the atoms of $-N_3$ and ring segment as independent rigid bodies. The total force and torque on these two rigid bodies were computed as the sum of the forces on its constituent beads.

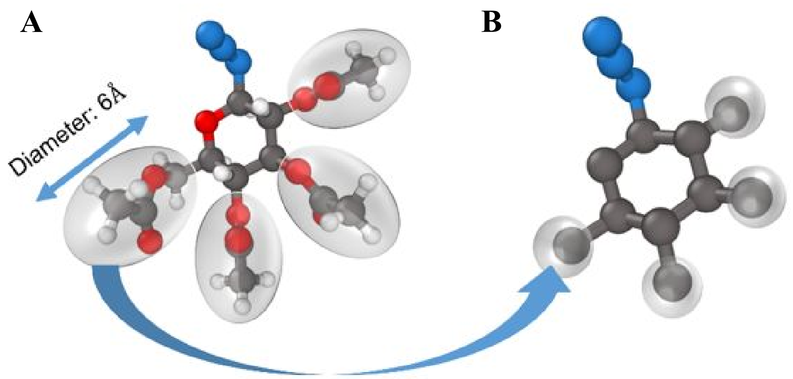

Supplementary Figure 29. (A) Structure of GluA molecule, with grey, red, and white beads representing the carbon, oxygen, and hydrogen atoms, and the blue beads showing the nitrogen atoms. (B) Coarse-grained model of GluA molecule. The blue and grey spheres represent N and R beads, respectively.

B. Simulation details

The nonbonded interactions between all segments take the form of a truncated and shifted Lennard-Jones potential:

$$U_{LJ} = \begin{cases} 4\varepsilon\left[\left(\frac{\sigma}{r}\right)^{12} - \left(\frac{\sigma}{r}\right)^{6}\right] - U_{cut} & r < r_{cut} \\ 0 & r > r_{cut} \end{cases} \quad (1)$$

Here, σ was the diameter of one bead, r_{cut} was the cutoff distance, and $U_{cut} = 4\varepsilon[(\sigma / r_{cut})^{12} - (\sigma/r_{cut})^{6}]$. In our simulation, σ corresponds to a coarse-grained molecule bead and is of the order of $\sigma = 0.1\, nm$. $\varepsilon = k_{\mathrm{B}}T$ sets the energy scale, where k_{B} is the Boltzmann constant. The time unit in our simulation was set to $\tau = \sqrt{\varepsilon/\sigma^2}$.

In our simulations, the repulsive interactions between R-N particles are captured by a truncated and shifted LJ potential (r_{cut} = 1.12 σ_0) with ε_{RN} = 1.0 ε. The attractions between N-N and R-R particles were characterized by a full range LJ potential ($r_{cut} = 2.5\ \sigma_0$) with strength $\varepsilon_{NN} = 4\ \varepsilon$ and $\varepsilon_{RR} = 1\ \varepsilon$, respectively.

The neighboring segments were connected by bonded interactions which was represented by a linear spring potential:

$$U_b = \frac{1}{2}k(r - r_0)^2 \quad (2)$$

where k = 5.0 ε was the spring coefficient, and $r_0 = 1\ \sigma$ was the equilibrium bond distance between two beads.

We used the open-source software LAMMPS[8] for all MD simulations based on the above force fields. All simulations were carried out by the velocity Verlet algorithm with a time step of $0.002\tau_0$. During the simulation, the temperature was controlled by the Nose–Hoover thermostat with NVT ensemble.

C. Influence of bead concentration

In experiment, the dilute solution of GluA molecules was cooled down in a refrigerator to synthesize the nanofibers with orientation and helix. The concentration of GluA molecules is 8 mg/ml with the molecular weight of 373 g/mol, yielding a concentration of $1.0 \times 10^{-4}\sigma^{-3}$ in our simulations. Direct simulation of system in such low concentrations are extremely slow. Many experiments and theories have confirmed a two-step crystallization process of organic molecules in dilute solutions during cooling. The first step is the cooling induced liquid-liquid phase separation[9-11], generating a metastable dense droplet, see Figure 35a-b. Subsequently, crystallization and self-assembly processes were initiated inside the dense droplets (Figure 35c). Therefore, rather than constructed nanofiber structure directly from the dilute solution, the solutes first form dense liquid droplets, from which the order self-assembled clusters appear and grows toward helix nanofibers. Here, we performed simulations of 512 molecules within a simulation box of the size $45\,\sigma \times 52\,\sigma \times 67\,\sigma$, yielding a concentration of $4.0 \times 10^{-2}\sigma^{-3}$ to mimic dense liquid phases, see Figure S35e.

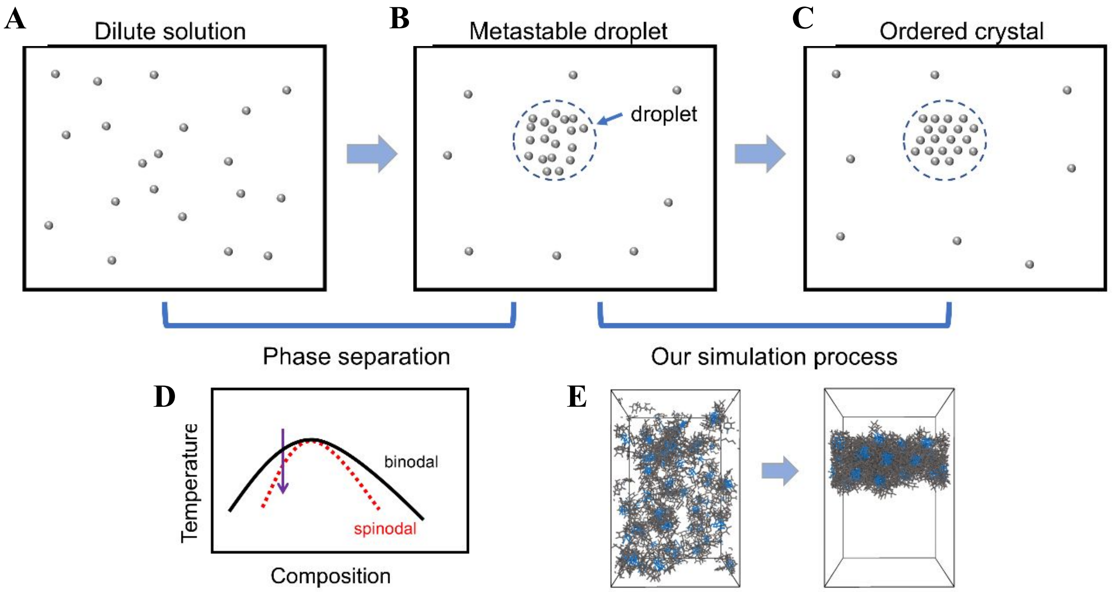

Supplementary Figure 30. The kinetic pathway leading from dilute solution to ordered crystals: (A) dilute solution, (B) dense metastable droplet after liquid-liquid phase separation, (C) ordered clusters of solute molecules. (D) Illustrative representation of temperature-composition phase diagram of molecules in solution. The black solid and the red dotted lines represent binodal and spinodal lines. The purple arrow shows the temperature quench that yields the phase separation. (E) Self-assembly of GluA molecules into helical cylinder in our simulation, where bule and grey spheres correspond to N and R beads.

D. Influence of temperature

The system was first heated to a reduced temperature T = 5.0 for 1000 τ to release any possible tensions. Then, the system was cooled down within 1000 τ to 0.8, 1.0, 1.2, 1.4 respectively. Subsequently, the simulations were run for 200000 τ to reach equilibrium at corresponding temperatures, see Figure 36. One can see that the GluA molecules aggregated to form irregular large clusters at different temperatures 0.8, 1.0 and 1.2. At T = 1.4, the molecules aggregated to form a cylindrical cluster. Meanwhile, at T = 1.6, a quasi-spherical cluster was observed.

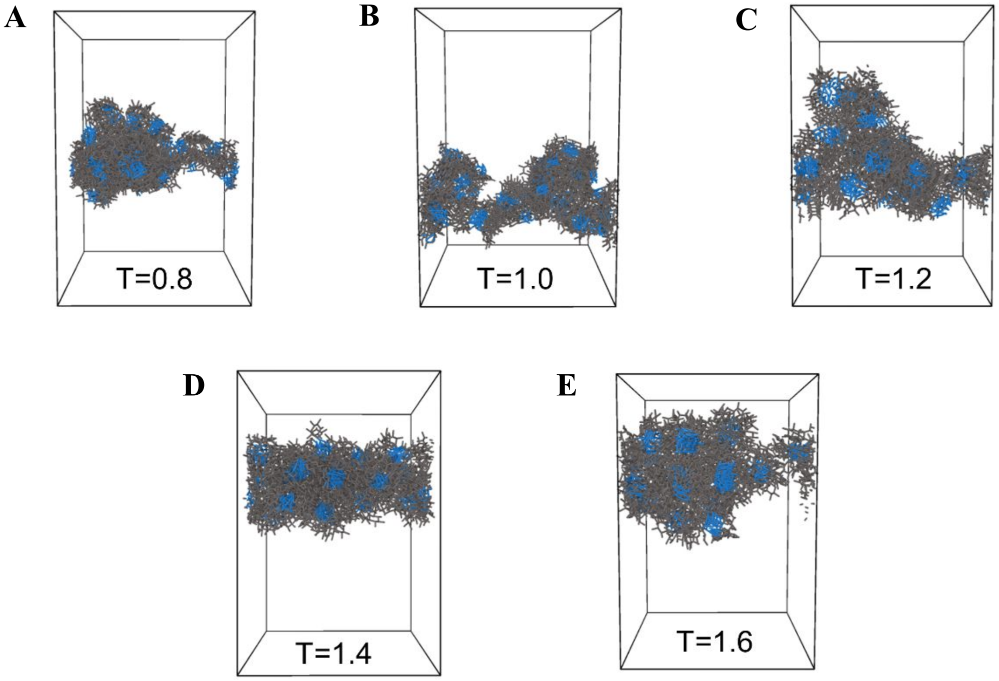

Supplementary Figure 31. Snapshots of equilibrium configurations of GluA molecules at reduced temperature T of (A) 0.8, (B) 1.0, (C) 1.2, (D) 1.4, (E) 1.6. The N and R beads are presented by bule and grey, respectively.

E. Influence of cooling rate

Furthermore, we investigated the impact of the cooling rate on the final structure of the molecules. As shown in Figure 37, we cooled the solution from temperature T = 5.0 to T = 1.4 within 100 τ, 1000 τ, and 10000 τ, generating the cooling rate of $4.2 \times 10^{-2}\ \tau^{-1}$, $4.2 \times 10^{-3}\ \tau^{-1}$, $4.2 \times 10^{-4}\ \tau^{-1}$, respectively. It is clearly shown that the GluA molecules formed a cylindrical cluster at three cooling rates. This indicated the limited influence of the cooling rate on the final structure of molecule.

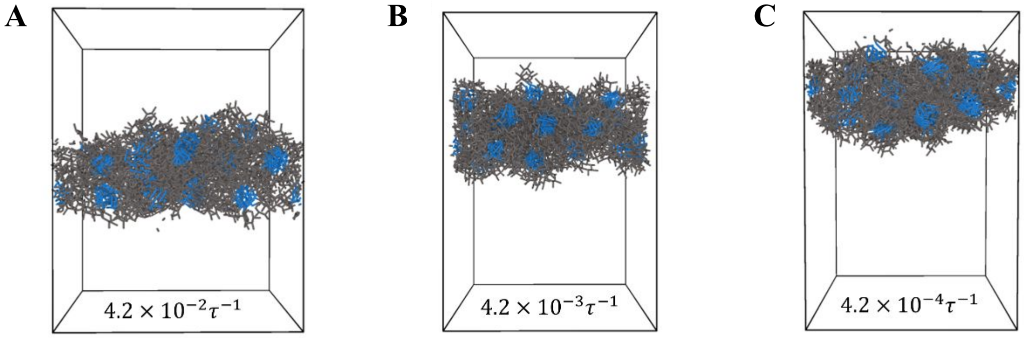

Supplementary Figure 32. The final state of the system with different cooling rates: (A) $4.2 \times 10^{-2}\ \tau^{-1}$, (B) $4.2 \times 10^{-3}\ \tau^{-1}$, (C) $4.2 \times 10^{-4}\ \tau^{-1}$.

Additional Figures

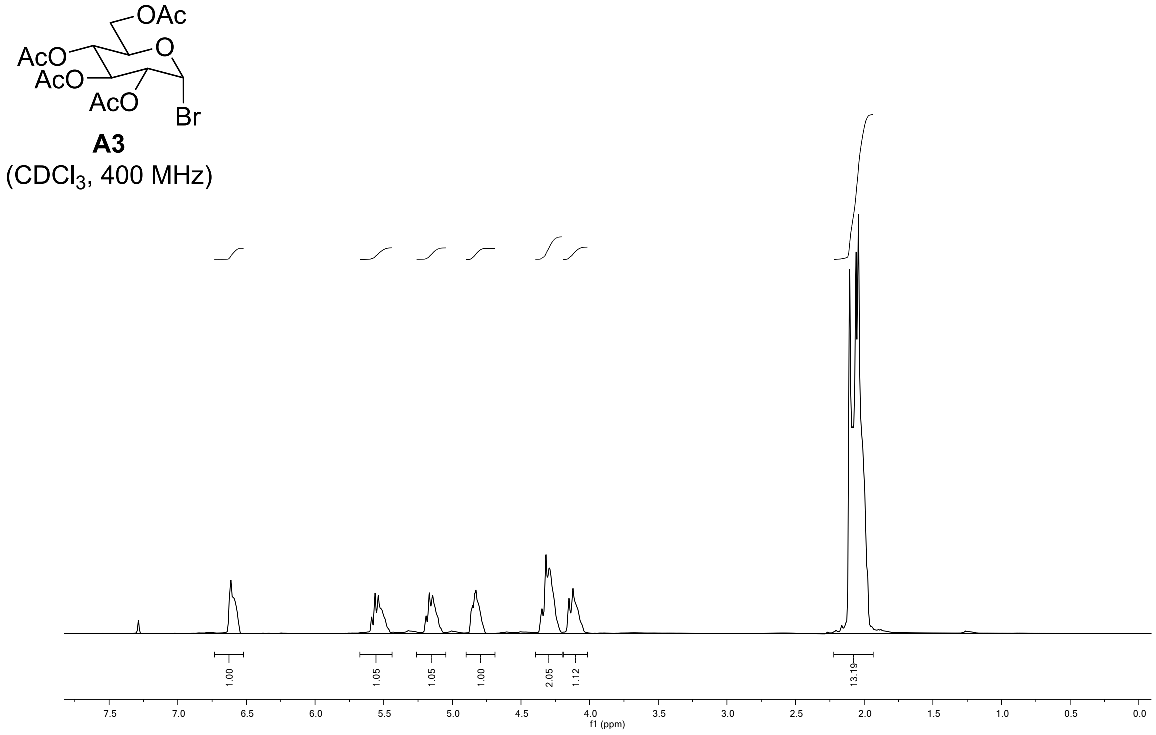

Supplementary Figure 33. ^{1}H NMR spectra of A3.

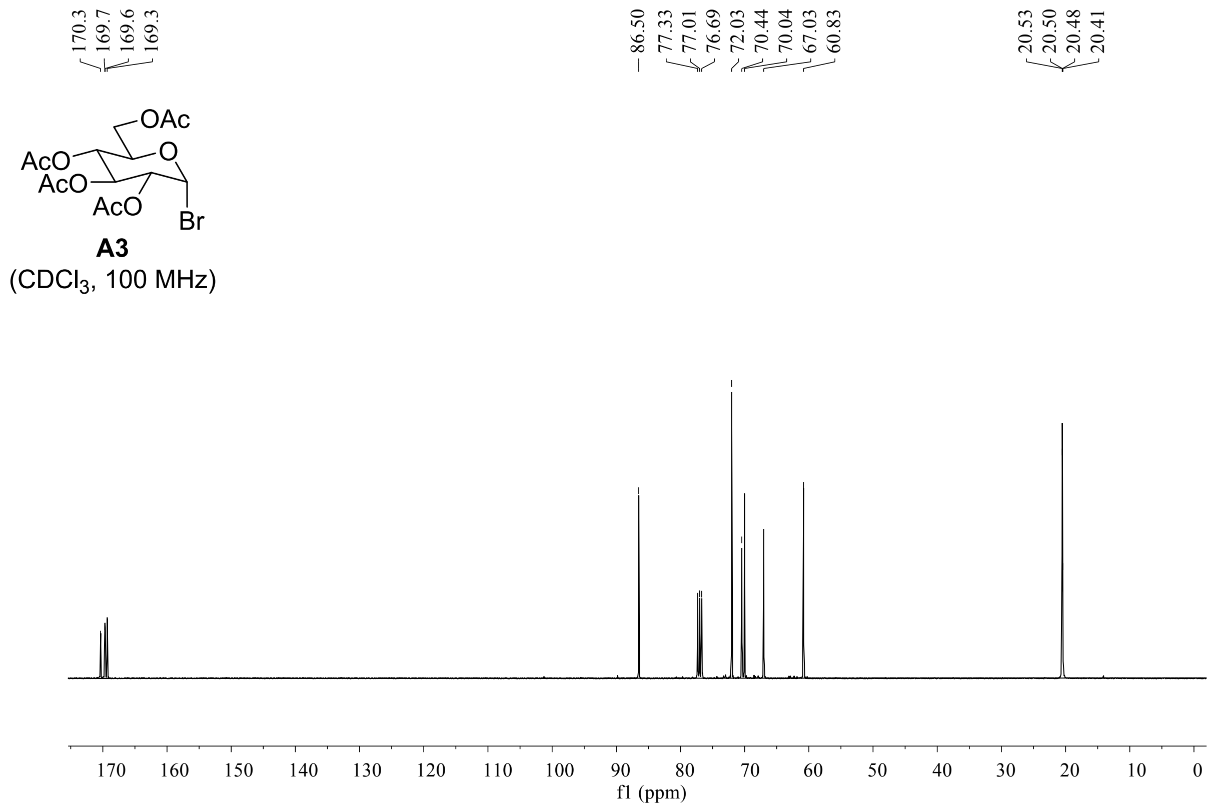

Supplementary Figure 34. ^{13}C NMR spectra of A3.

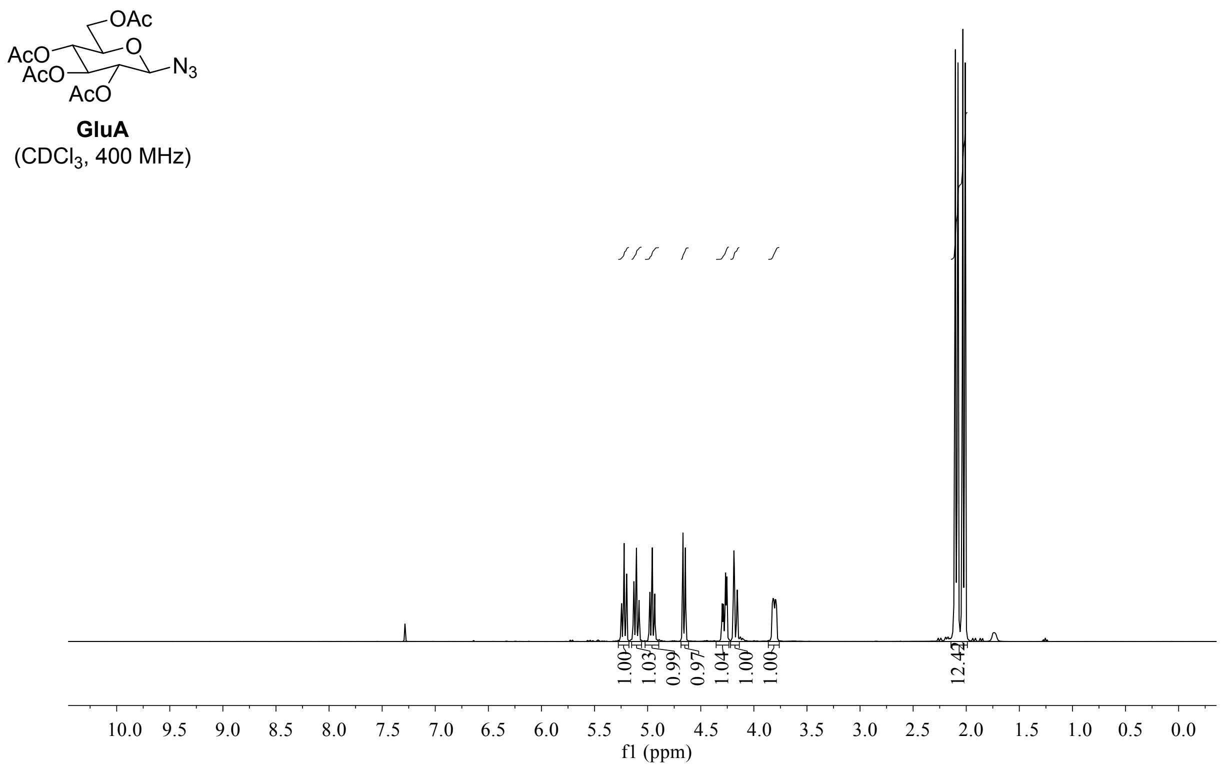

Supplementary Figure 35. ^{1}H NMR spectra of GluA.

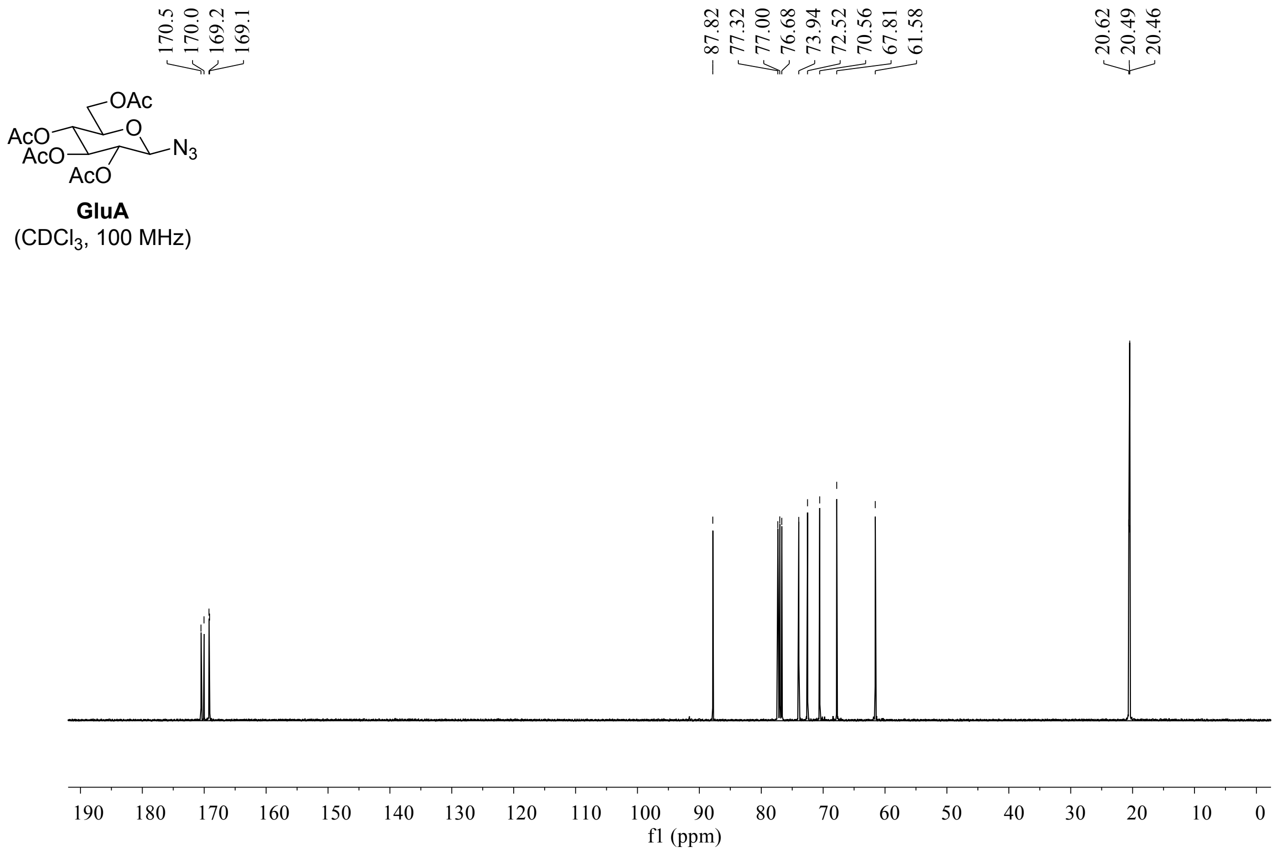

Supplementary Figure 36. ^{13}C NMR spectra of GluA.

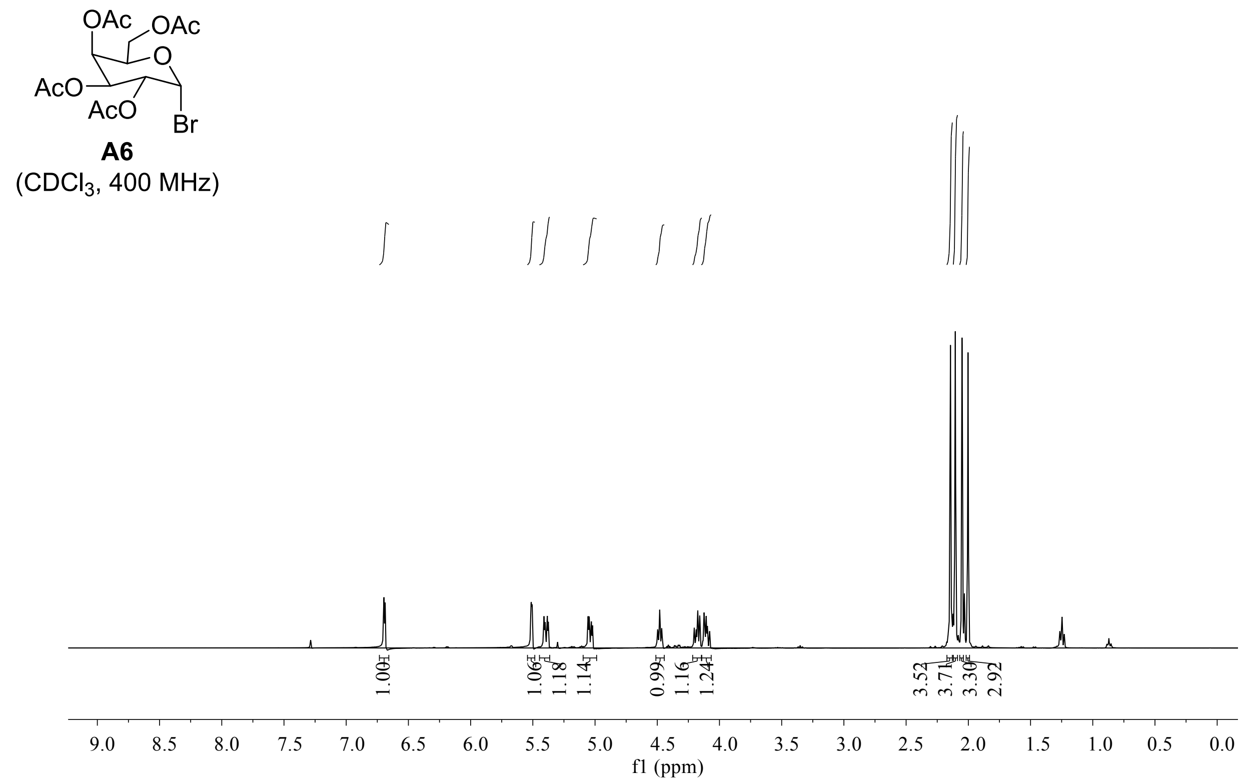

Supplementary Figure 37. ^{1}H NMR spectra of A6.

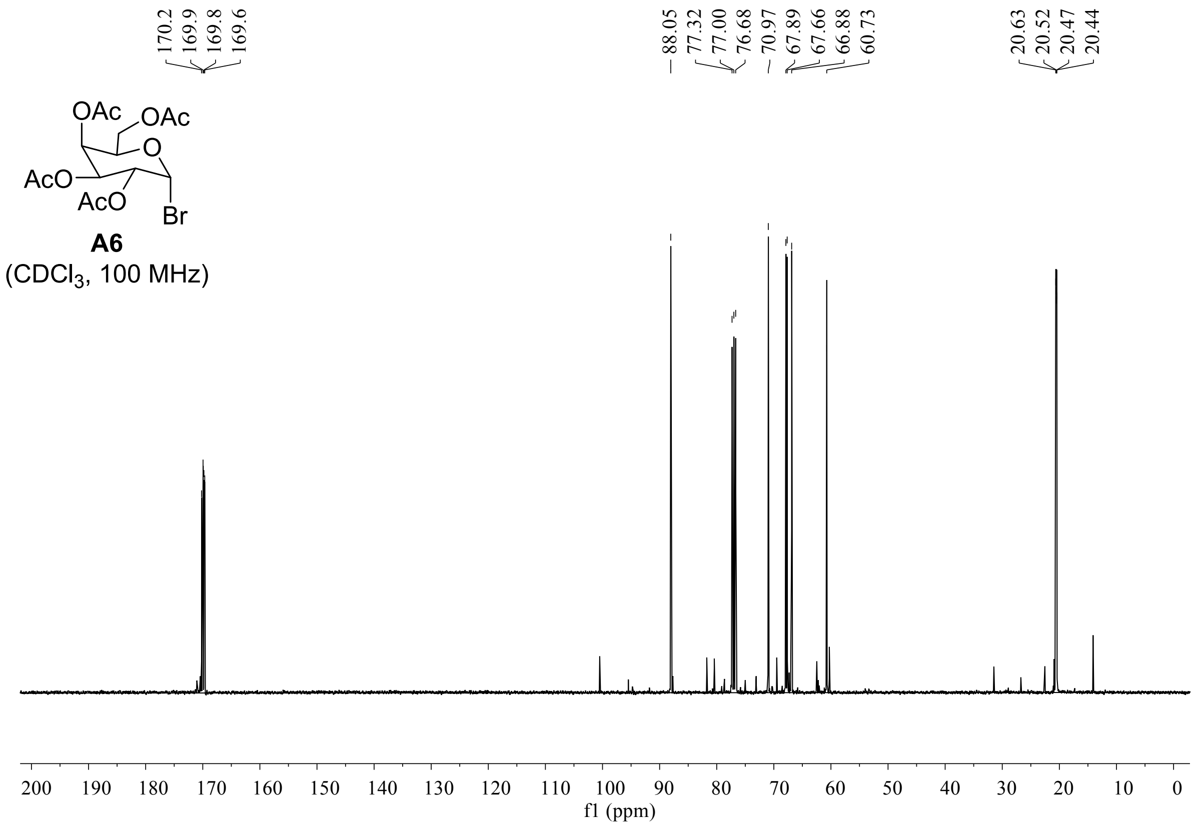

Supplementary Figure 38. ^{13}C NMR spectra of A6.

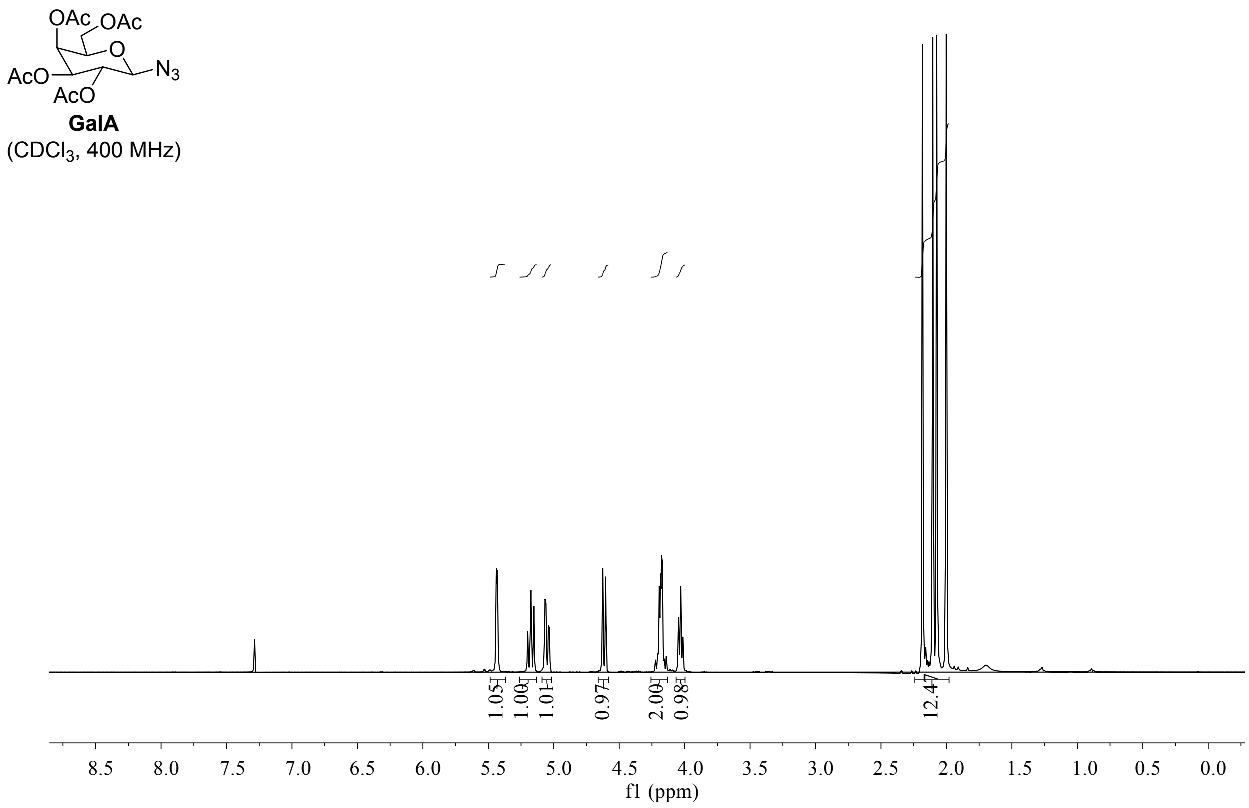

Supplementary Figure 39. ^{1}H NMR spectra of GalA.

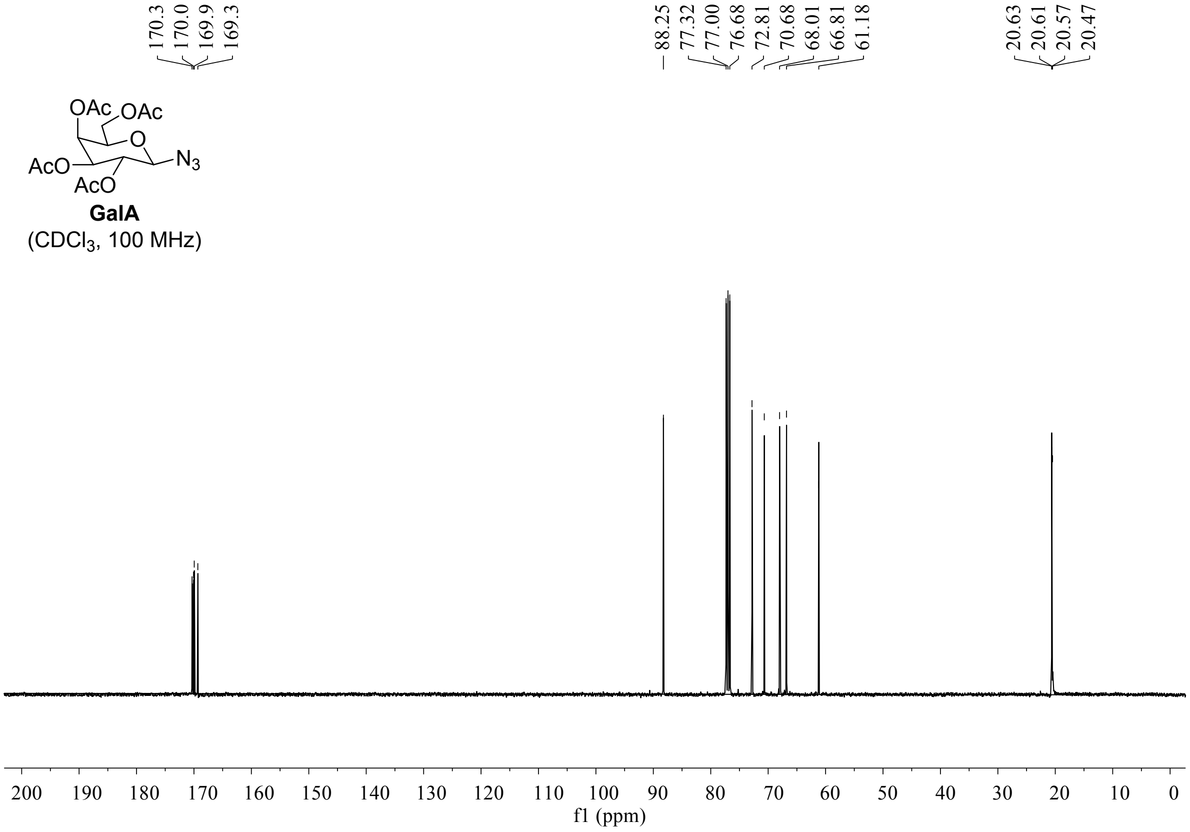

Supplementary Figure 40. ^{13}C NMR spectra of GalA.

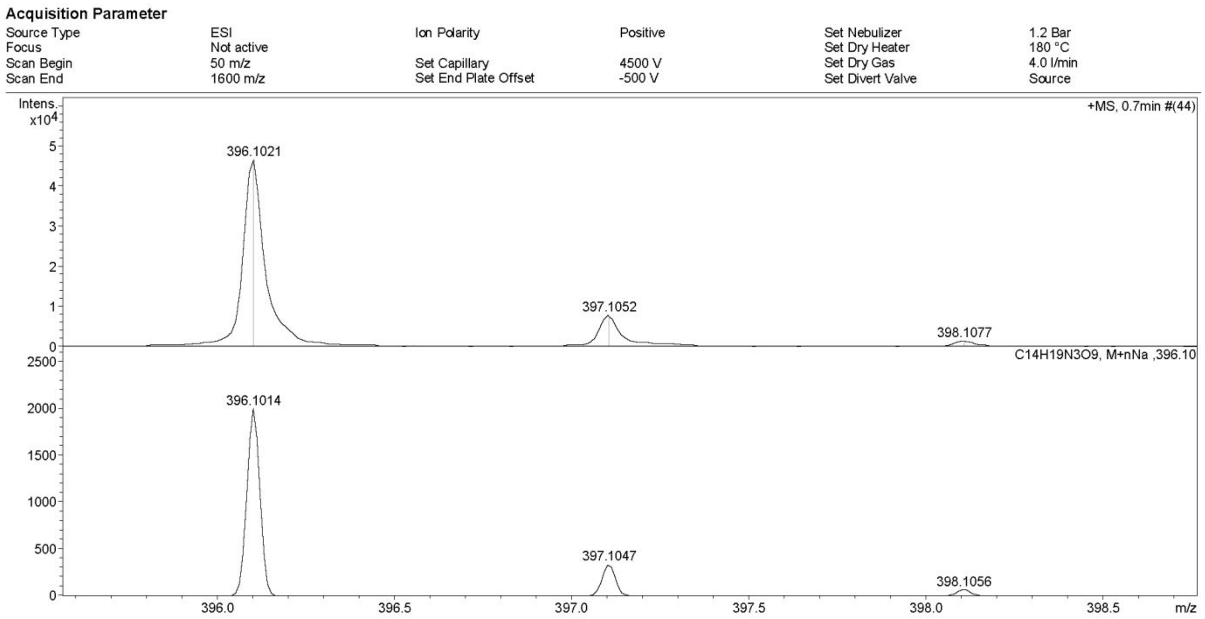

Supplementary Figure 41. ESI-HRMS analysis of GluA.

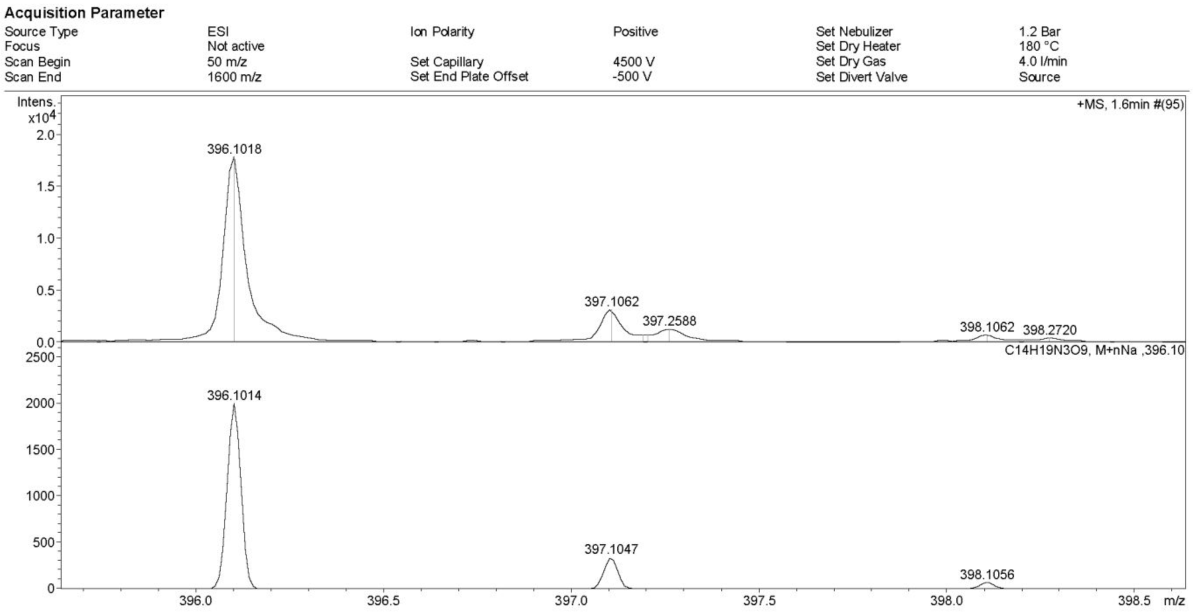

Supplementary Figure 42. ESI-HRMS analysis of GalA.

Reference

(1) Bruker, APEX4 v2021.4-0, SAINT vB.408. *Bruker AXS Inc. Madison*. **2021**.

(2) Krause, L.; Herbst-Irmer, R.; Sheldrick, G. M.; Stalke, D., Comparison of silver and molybdenum microfocus X-ray sources for single-crystal structure determination. *J. Appl. Cryst.* **2015,** 48, 3-10.

(3) Bruker, XPREP 2014/2. *Bruker AXS Inc. Madison*. **2014**.

(4) Sheldrick, G. M., SHELXT– Integrated space-group and crystal-structure determination. *Acta Cryst. A*. **2015,** 71, 3-8.

(5) Sheldrick, G. M., Crystal structure refinement with SHELXL. *Acta Cryst. C Structural Chemistry*. **2015,** 71, 3-8.

(6) Spackman, P. R.; Turner, M. J.; Mckinnon, J. J.; Wolff, S. K.; Grimwood, D. J.; Jayatilaka, D.; Spackman, M. A., CrystalExplorer: a program for Hirshfeld surface analysis, visualization and quantitative analysis of molecular crystals. *J. Appl. Cryst.* **2021,** 54, 1006-1011.

(7) Temelkoff, D. P.; Norris, P.; Zeller, M., 2,3,4,6-Tetra-O-acetyl-[beta]-d-glucopyranosyl azide. *Acta Cryst. E*. **2004,** 60, o1975-o1976.

(8) Grest, G. S.; Kremer, K., Molecular dynamics simulation for polymers in the presence of a heat bath. *Physical Review A*. **1986,** 33, 3628-3631.

(9) Gliko, O.; Neumaier, N.; Pan, W.; Haase, I.; Fischer, M.; Bacher, A.; Weinkauf, S.; Vekilov, P. G., A Metastable Prerequisite for the Growth of Lumazine Synthase Crystals. *Journal of the American Chemical Society*. **2005,** 127, 3433-3438.

(10) Yao, Y.; Tang, Q.; Rosenfeldt, S.; Krüsmann, M.; Karg, M.; Zhang, K., Tuning Sugar‐Based Chiral and Flower‐Like Microparticles. *Small*. **2021,** 17.

(11) Erdemir, D.; Lee, A. Y.; Myerson, A. S., Nucleation of Crystals from Solution: Classical and Two-Step Models. *Accounts of Chemical Research*. **2009,** 42, 621-629.

Other Supplementary Materials

Supplementary Movie 1

Bending-to-straightening behavior on the starting end. Scale bar 100 µm.

Supplementary Movie 2

Bending-to-straightening behavior on the stacking end. Scale bar 100 µm.

Supplementary Movie 3

Bending-to-straightening behavior among local crystallization areas 1. Scale bar 100 µm.

Supplementary Movie 4

Bending-to-straightening behavior among local crystallization areas 2. Scale bar 100 µm.
